# Auditing widely used biomolecular benchmarks reveals systematic data inconsistencies

**DOI:** 10.1039/d6sc01799a

**Published:** 2026-07-22

**Authors:** Maximilian G. Schuh, Aleksandra Daniluk, Stephan A. Sieber

**Affiliations:** a TUM School of Natural Sciences, Department of Biosciences, Chair of Bioorganic Chemistry, Center for Functional Protein Assemblies, Technical University of Munich (TUM) Ernst-Otto-Fischer-Str. 8 85748 Garching Germany stephan.sieber@tum.de

## Abstract

Machine learning accelerates molecular discovery and relies heavily on standardised benchmark datasets to evaluate computational performance. To learn transferable structure–activity relationships (SARs), models must be trained on datasets that accurately reflect chemical and biological realities without introducing artificial redundancies. Although standardised benchmarks are ubiquitous, the integrity of their underlying data is often assumed rather than rigorously verified. Currently, there is a lack of systematic and quantitative assessments of hidden data leakage and structural inconsistencies across widely used biomolecular benchmarks. Here, we demonstrate that several prominent chemical and biochemical benchmarking suites have pervasive cross-split contamination, unresolved label conflicts, and severe structural redundancies. By auditing over fifty dataset configurations, we reveal that tasks previously considered robust evaluation environments often have hidden flaws, particularly in drug–target interaction datasets with extensive protein overlap. Controlled noise-injection experiments show that these artefacts can systematically bias benchmark metrics. A complementary counterfactual leaderboard analysis further shows that leading-model conclusions can change when the audited composition of the test set is altered, particularly when label conflicts are enriched. These findings suggest that many leaderboard and leading-model conclusions should be interpreted in light of benchmark composition and data quality, rather than as automatic evidence of robust methodological superiority. Our findings highlight the importance of auditing evaluations, reporting chemically meaningful test-set composition, and using leakage-resistant data splits to accurately measure model generalisability. Moving from uncritical leaderboard optimisation to rigorous dataset auditing will yield more reliable computational tools. Ultimately, this will ensure that artificial intelligence models can be reliably translated into real-world applications in therapeutic design.

## Abstract 2

Machine learning accelerates molecular discovery and relies heavily on standardised benchmark datasets to evaluate computational performance.

## Graphical abstract

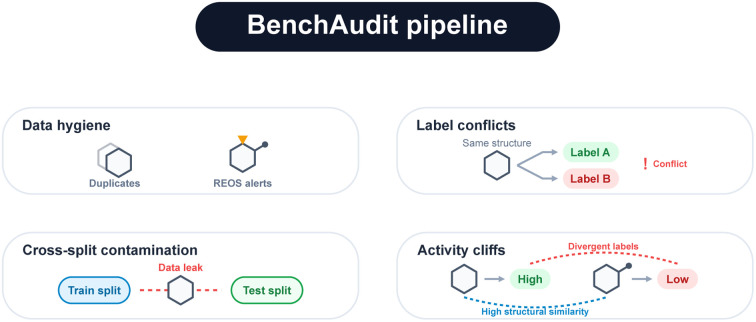

## 1 Introduction

Benchmarks have catalysed rapid advances in biomolecular machine learning (ML) by standardising tasks and enabling direct comparison of models.^1–6^ However, the validity of these comparisons relies fundamentally on the fidelity of the underlying datasets.^7–9^ Subtle but consequential data issues, such as duplicate records, inconsistent annotations, misparsed structures, or inadvertent train–test leakage, can inflate performance estimates and reward models that memorise artefacts rather than infer transferable SARs.^7,9–13^ Despite growing concerns within the community regarding the reliability of widely used benchmarks, a rigorous, systematic, and quantitative assessment remains absent.^14–16^ Evidence of benchmark deficiencies has already emerged in protein–ligand binding affinity datasets, including PDBbind/CASF and various reorganised PDBbind splits.^17–19^ However, existing analyses are typically confined to individual datasets, specific prediction endpoints, or particular splitting strategies, preventing a broader understanding of benchmark robustness and validity.

**Fig. 1:**
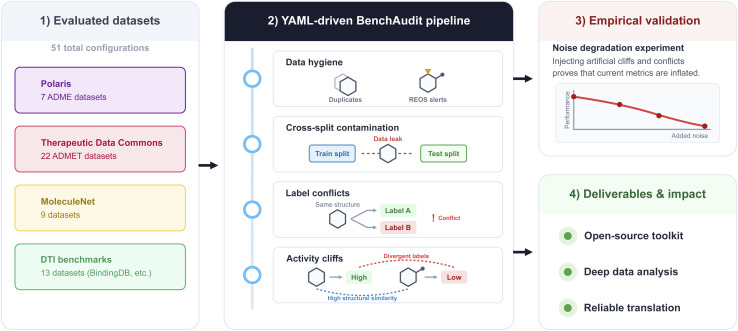
Overview of the study design and auditing framework. (1) Benchmark collections analysed in this study, comprising 51 benchmark configurations from Polaris, Therapeutics Data Commons, moleculeNet, and drug–target interaction datasets. (2) Configuration-driven auditing workflow illustrating the modular pipeline, including data hygiene, cross-split contamination analysis, label-conflict detection, and activity-cliff identification. (3) Empirical validation through controlled noise-injection experiments to quantify the impact of common data artefacts on model performance. (4) Key outcomes of this work, including an open-source auditing toolkit, comprehensive benchmark analyses, and recommendations for more reliable benchmark evaluation and translation into real-world applications.

Without such an assessment, safeguarding dataset integrity remains difficult, hindering meaningful modelling innovation (Fig. 1).

To address this gap, we introduce a reproducible, configuration-driven audit framework designed to systematically evaluate and strengthen the integrity of chemo- and biochemical ML benchmarks. Our central contribution is the formalisation of data quality risks and leakage mechanisms into a clearly defined and operational set of audit criteria. By instantiating these criteria, the framework produces a multi-dimensional integrity report that assesses each benchmark along the full pipeline, from fundamental curation properties to advanced cross-split leakage diagnostics.

We apply this framework to the most widely adopted benchmark suites in the field, including Polaris,^20–22^ Therapeutics Data Commons (TDC),^23,24^ MoleculeNet,^25^ and several drug–target interaction (DTI) datasets,^26–32^ collectively accounting for more than 10 000 citations. Despite their prominence and broad community trust, our analysis uncovers pervasive cross-split contamination, structural redundancy, and label inconsistencies across a majority of the evaluated datasets. This work distinguishes itself from recent curation studies focused on PDBbind or protein–ligand binding affinity.^17–19^ Our framework is designed as a general benchmark auditing layer rather than a dataset-specific correction pipeline. Through controlled noise-injection experiments, we further demonstrate that such artefacts can measurably and systematically inflate reported model performance, thereby distorting comparative evaluations.

To enable immediate adoption and practical impact, we release our flexible, YAML-driven auditing toolkit as an open-source resource, together with deep data diagnosis and reporting. By embedding quantitative auditing directly into the benchmark lifecycle, our framework promotes more reliable model development and helps ensure that reported scientific conclusions reflect genuine generalisation rather than inadvertent dataset exploitation.

## 2 Study design

To systematically evaluate benchmark quality, we define the following audit criteria covering hygiene, leakage, and label consistency. These criteria are intended as diagnostic indicators rather than automatic judgements of benchmark invalidity. For example, activity cliffs may reflect meaningful SARs, and ligand reuse in DTI datasets can be scientifically legitimate when the target differs. The purpose of *BenchAudit* is therefore to make such structural properties explicit, comparable, and reproducible, so that benchmark users can judge whether a split is appropriate for a given generalisation claim.

### 2.1 Audit criteria

#### 2.1.1 Duplicate molecules and sequences

We define a duplicate molecule as two or more rows within the same split sharing the same cleaned simplified molecular-input line-entry system (SMILES) string. In the DTI setting, duplicates are also counted at the level of target sequences (identical amino-acid sequences), even if their ligands differ.^33^

#### 2.1.2 Contamination

Contamination refers to overlap between training (and optionally validation) data and the held-out test set. We quantify unique cleaned SMILES appearing in both partitions. In DTI analyses, contamination is computed for ligands, target sequences, and total ligand–target pairs shared across splits.

#### 2.1.3 Molecular similarity

We apply a consensus rule inspired by van Tilborg *et al.*^13^ A pair of molecules is considered similar if any of three measures exceeds the configured 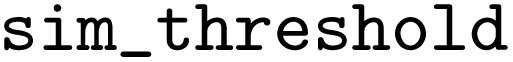 (default 0.9): (i) Tanimoto similarity of Morgan fingerprints (radius 2, 2048 bits); (ii) Tanimoto similarity of generic Murcko scaffolds; or (iii) normalised Levenshtein similarity of the SMILES strings.

#### 2.1.4 Nearest-neighbour similarity

To summarise distributional distance, the analyser computes the maximum Tanimoto similarity for each molecule in a query split (*e.g.*, test) against all molecules in a reference split (*e.g.*, train). We report the mean, standard deviation, and sample size of these nearest-neighbour scores (default: 50).

#### 2.1.5 Label conflicts

For classification, conflict occurs when the same cleaned SMILES appears with differing label tuples. For regression, a conflict is defined as a pair of rows sharing the same SMILES but having labels that differ by at least 3*σ* (where *σ* is the standard deviation of the training labels). This 3*σ* rule provides a scale-free notion of substantial disagreement.

#### 2.1.6 Activity cliffs

Activity cliffs are pairs of molecules with high similarity but divergent activity. Using the consensus similarity rule, the analyser identifies pairs exceeding the threshold; if their labels differ (classification) or differ by ≥3*σ* (regression), a cliff is recorded. These are counted separately for intra-split and cross-split events.

#### 2.1.7 Sequence-level conflicts in DTI analyses

We define a cross-split ligand–target pair conflict when the same combination of a cleaned ligand and a normalised target appears in multiple splits. We also track sequence multi-ligand reuse, where a target sequence appears with different ligands across splits, to provide a detailed view of potential information leakage.

#### 2.1.8 Sequence similarity

Sequence similarity is quantified using pairwise global alignment *via* the EMBOSS 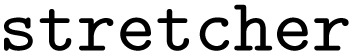 program.^34^ We identify the best alignment for each query sequence against a reference split and summarise the distribution of identity and similarity scores.

#### 2.1.9 Data provenance

We assess the documentation and provenance of each benchmark. This includes verifying whether the dataset is anchored in peer-reviewed sources, whether task definitions and label semantics are documented, and whether the splitting procedures are deterministic and programmatically reproducible.

### 2.2 Evaluated datasets

We applied these audit criteria to a comprehensive set of benchmarks sourced from three primary collections and standalone publications (Table 1).

**Table 1:** Benchmark inventory with source, source-local index, benchmark name, and reference

Source	No.	Name	Ref.
Polaris	1	Novartis-adme-novartis-cyp3a4-reg	20 and 22
Polaris	2	Polaris-adme-fang-hclint-1	20 and 21
Polaris	3	Polaris-adme-fang-hppb-1	20 and 21
Polaris	4	Polaris-adme-fang-perm-1	20 and 21
Polaris	5	Polaris-adme-fang-rclint-1	20 and 21
Polaris	6	Polaris-adme-fang-rppb-1	20 and 21
Polaris	7	Polaris-adme-fang-solu-1	20 and 21
TDC	1	BBB_Martins	23 and 24
TDC	2	Bioavailability_Ma	23 and 24
TDC	3	CYP1A2_Veith	23 and 24
TDC	4	CYP2C19_Veith	23 and 24
TDC	5	CYP2C9_Substrate_CarbonMangels	23 and 24
TDC	6	CYP2C9_Veith	23 and 24
TDC	7	CYP2D6_Substrate_CarbonMangels	23 and 24
TDC	8	CYP2D6_Veith	23 and 24
TDC	9	CYP3A4_Substrate_CarbonMangels	23 and 24
TDC	10	CYP3A4_Veith	23 and 24
TDC	11	Caco2_Wang	23 and 24
TDC	12	Clearance_Hepatocyte_AZ	23 and 24
TDC	13	Clearance_Microsome_AZ	23 and 24
TDC	14	HIA_Hou	23 and 24
TDC	15	Half_Life_Obach	23 and 24
TDC	16	HydrationFreeEnergy_FreeSolv	23 and 24
TDC	17	Lipophilicity_AstraZeneca	23 and 24
TDC	18	PAMPA_NCATS	23 and 24
TDC	19	PPBR_AZ	23 and 24
TDC	20	Pgp_Broccatelli	23 and 24
TDC	21	Solubility_AqSolDB	23 and 24
TDC	22	VDss_Lombardo	23 and 24
MoleculeNet	1	BACE	25
MoleculeNet	2	BBBP	25
MoleculeNet	3	ClinTox	25
MoleculeNet	4	ESOL	25
MoleculeNet	5	FreeSolv	25
MoleculeNet	6	HIV	25
MoleculeNet	7	Lipophilicity	25
MoleculeNet	8	SIDER	25
MoleculeNet	9	Tox21	25
DTI	1	BindingDB_cluster_cls	26
DTI	2	BindingDB_protein_cls	26
DTI	3	BindingDB_random_cls	26
DTI	4	BindingDB_scaffold_cls	26
DTI	5	BioSNAP_cluster_cls	27
DTI	6	BioSNAP_protein_cls	27
DTI	7	BioSNAP_random_cls	27
DTI	8	BioSNAP_scaffold_cls	27
DTI	9	Human_protein_cls	28
DTI	10	Human_random_cls	28
DTI	11	Human_scaffold_cls	28
DTI	12	PDBBindv2016_reg	29 and 30
DTI	13	PDBBindv2020_reg	31 and 32

#### 2.2.1 Polaris benchmarks

The Polaris platform provides benchmarks covering the “adme-fang” panel and a Novartis CYP3A4 regression dataset.^20–22^ Polaris manages data retrieval and evaluation internally to ensure consistent baseline modelling.

#### 2.2.2 TDC benchmarks

The TDC collection extends coverage towards absorption, distribution, metabolism, excretion, and toxicity (ADMET) endpoints, accessed *via* the TDC interface with random and Bemis–Murcko scaffold splits. The panel spans endpoints including CNS penetration, bioavailability, CYP450 activity, and metabolic stability.^23,24^

#### 2.2.3 MoleculeNet benchmarks

The MoleculeNet suite provides reference datasets for classification (BACE, BBBP, ClinTox, HIV, SIDER, and Tox21) and regression (ESOL, FreeSolv, and Lipophilicity). Each dataset supplies predefined splits. Multi-task classification datasets (ClinTox, SIDER, and Tox21) construct label vectors from multiple binary label columns.^25^

The DTI benchmarks characterise datasets pairing ligand SMILES with protein sequences. Four major families are included.

#### 2.2.4 BindingDB-derived benchmarks

These classification datasets support random, scaffold-based, cluster-based, and protein-based splits, providing binary interaction labels for ligand–target pairs.^26^

#### 2.2.5 BioSNAP benchmarks

BioSNAP benchmarks mirror the BindingDB format with analogous split types, facilitating systematic comparisons of leakage and redundancy.^27^

#### 2.2.6 Human DTI benchmarks

These datasets focus on binary interactions involving human proteins, providing random, scaffold, and protein splits.^28^

#### 2.2.7 PDBbind-derived benchmarks

The PDBbind v2016 and v2020 releases supply regression tasks for binding affinity prediction, including ligand SMILES, protein sequences, and continuous affinity labels.^29–32^

## 3 Results and discussion

**Fig. 2:**
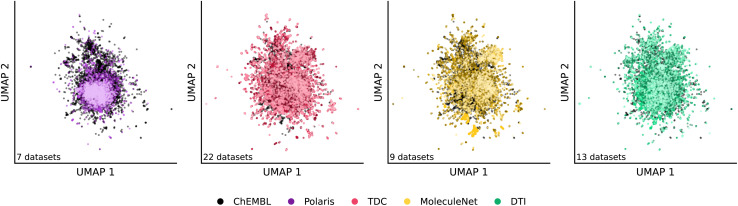
Chemical space visualisation *via* UMAP. The black points indicate 10 000 random samples from ChEMBL-36.^35^ The coloured points indicate the benchmark chemical space, showing where molecules lie within the global chemical space. 1000 molecules were sampled for each dataset and visualised in a different shade.

**Fig. 3:**
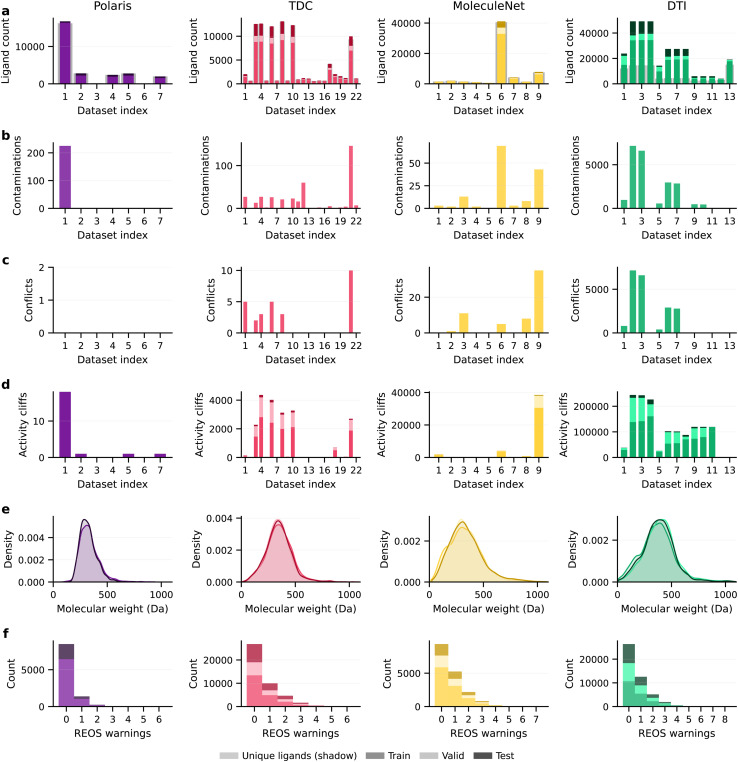
Ligand-space benchmark diagnostics across dataset families. The grid reports per-dataset quality and distribution checks for Polaris, TDC, MoleculeNet, and DTI benchmarks, with the dataset index progressing along each *x*-axis. (a) Split sizes with a unique-ligand reference shadow: stacked train/valid/test ligand counts are shown against a black unique-ligand shadow bar. (b) Ligand contamination from train/valid into test. (c) Severe cross-split conflicts (test → train + valid, ≥3*σ* criterion). (d) Activity cliff counts, stacked by intra-train/valid, cross-split, and intra-test components. (e) Molecular weight distributions by split. (f) REOS warning count distributions by split (stacked histograms). Train/valid/test use consistent shade coding within each benchmark family, and subtle horizontal grid lines aid row-wise visual comparison.

We applied the pipeline to a total of 51 benchmark configurations (7 Polaris, 22 TDC, 9 MoleculeNet, and 13 DTI; Table 1). Evaluating these configurations against our defined criteria, we first examined their chemical space coverage (Fig. 2 and S1–S4). We observed that most benchmark suites span a relatively broad chemical space compared to ChEMBL.^35^ The primary exception is Polaris, which appears more focused. A plausible explanation is its comparatively smaller number of molecules and its more narrowly defined objective, absorption, distribution, metabolism, and excretion (ADME) prediction, rather than broader, general-purpose benchmarking. This is in contrast to the others, which all contain significantly more ligand–measurement data points (Fig. 3a).

### 3.1 Hidden data leakage and conflicts

We investigated the number of samples contaminating the test set (Fig. 3b), *i.e.*, cases where the same molecule also appears in the training or validation split. Overall, such contaminations are widespread and occur across essentially all benchmark suites. They are present in the vast majority of datasets, indicating that split leakage is a recurring rather than isolated issue. Polaris exhibits contamination in only a single dataset, which is comparatively large and therefore more prone to repeated compounds across splits. In contrast, contaminations occur frequently in the DTI benchmarks, which are expected because we quantify overlap only in one modality (the drug) rather than in the full drug–target pair. We next examined conflicts of ligand–measurement pairs across splits (Fig. 3c), defined as cases where the same molecule is associated with different labels. Here, Polaris shows no conflicts, whereas both TDC and MoleculeNet contain several. For the DTI benchmarks, apparent conflicts are largely explained by the second modality (the protein), as the same drug can legitimately exhibit different outcomes against different targets. These contaminations and conflicts likely stem from inhomogeneous sources and artefacts introduced during data aggregation and harmonisation. More rigorous data processing and cleaning would mitigate these issues and should provide more realistic estimates of predictive performance for ML/deep learning (DL) approaches. Although performance metrics on cleaned datasets may appear “worse”, they are more meaningful because they reduce the risk of models being “confused” by contradictory labels for the same molecule across splits.

We further quantified activity cliffs across all datasets (Fig. 3d), a property that is particularly relevant for settings such as SAR studies, where models are expected to capture subtle structural changes and respond appropriately to sharp shifts in activity. This metric also may carry an implicit interpretation: a higher prevalence of cliffs could reflect increased heterogeneity in the underlying data generation process, for instance, when measurements from multiple sources or experimental conditions are aggregated. Such aggregation can introduce additional apparent cliffs because nominally similar experiments performed in different environments may yield different outcomes. Importantly, this relationship is not causal, and the presence of activity cliffs is not inherently undesirable, as many tasks explicitly aim to model this challenging regime.^13^ Activity cliffs are well-established manifestations of SAR discontinuity and often represent valuable, information-rich medicinal-chemistry examples (Fig. S5). In our analysis, Polaris exhibits comparatively few activity cliffs, whereas TDC and MoleculeNet contain a substantial number. The DTI benchmarks show the highest prevalence, which is consistent with our earlier observations and may be related to assessing overlap primarily on the ligand side. To further contextualise these findings in terms of chemical space, we computed the molecular weight distributions for all datasets (Fig. 3e). Across benchmarks and splits, the distributions are broadly similar, with an average molecular weight of approximately 300 Da. Notably, Polaris displays a comparatively narrow distribution, whereas the DTI datasets span a broader range of molecular weights.

Normalising artefact counts by dataset size reveals a clear hierarchy of structural vulnerability across benchmarks. Among all evaluated datasets, the highest contamination rate is observed for TDC dataset 12 (Clearance_Hepatocyte_AZ) at 4.95%, while MoleculeNet dataset 3 (ClinTox) exhibits the highest severe conflict rate at 0.74%, and MoleculeNet dataset 1 (BACE) exhibits the highest cliff density at 0.175%. Collectively, these relative metrics demonstrate that many datasets contain non-trivial structural deficiencies that can meaningfully affect evaluation outcomes.

Beyond these internal structural deficiencies, the robustness of evaluation is further impacted by cross-split data leakage. Accordingly, we observed substantial differences in the mean nearest-neighbour extended-connectivity fingerprint (ECFP) Tanimoto similarity between the test set and the combined training and validation sets across benchmark categories. When aggregated within each category, the mean similarity was 0.494 for Polaris, 0.559 for TDC, 0.589 for MoleculeNet, and 0.779 for DTI. Under this nearest-neighbour definition, these values imply that DTI benchmarks more often contain test ligands that are highly similar to ligands already observed during training, whereas the other categories exhibit markedly lower cross-split similarity on average.

Next, we evaluated the chemical “quality” represented in the datasets by quantifying rapid elimination of swill (REOS)/Dundee warnings across splits (Fig. 3f).^36^ These warnings flag substructures that are often considered undesirable in medicinal chemistry and therefore provide a coarse but informative proxy for compound plausibility and applicability. Polaris again stands out, exhibiting only a few ligands with any warnings and almost none with more than two flags. In contrast, all other benchmark categories show substantially higher numbers of REOS warnings, including a noticeable fraction of molecules with multiple alerts. This trend is particularly pronounced for MoleculeNet, which contains a considerable number of compounds that would typically violate traditional drug-likeness criteria.

### 3.2 Analysis of drug–target pairs

**Fig. 4:**
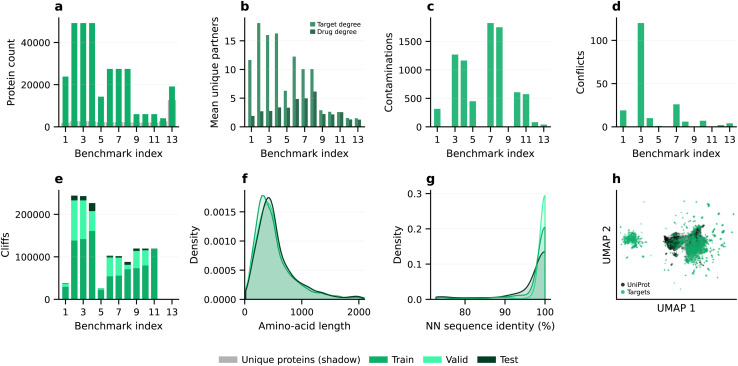
DTI pair-space and target-space diagnostics across benchmark datasets. The dashboard summarises split composition, leakage risk, and target-space structure for each DTI benchmark (columns grouped by dataset index). (a) Protein counts per dataset: total proteins are shown in base green bars, with unique proteins overlaid as a wider grey shadow for visual comparison. (b) Mean train + valid promiscuity: average unique drugs per target (light green) and unique targets per drug (dark green). (c) Target contamination from train/valid into test. (d) Cross-split conflict counts (pair/sequence inconsistency summary). (e) Activity cliff counts, stacked by intra-train/valid, cross-split, and intra-test components. (f) Amino acid length distributions by split (train/valid/test shades). (g) Nearest-neighbour sequence identity distributions for valid → train, test → train, and test → train + valid comparisons. (h) Protein-space UMAP (ESM C^37^): black points indicate UniProt background (10 000 random samples), and green points indicate benchmark target proteins, showing where benchmark targets lie within the global protein space.

When moving from single-modality molecular benchmarks to the DTI setting, a distinct structural pattern emerges. The DTI benchmarks stand out not only because of the large number of data points on the drug side (Fig. 3a), but also due to the substantial volume on the target side (Fig. 4a). However, these absolute numbers must be interpreted in context, as the number of unique entities is comparatively small relative to other benchmark categories. This is inherent to the DTI setup, where assays are typically performed on a fixed protein target that is screened against multiple candidate compounds, such as potential inhibitors.

The interaction statistics (Fig. 4b) reflect this structure: it is common to observe roughly a dozen unique drugs per protein target, whereas a single molecule is usually associated with at most a handful of distinct proteins (often fewer than six). At the same time, the DTI benchmarks exhibit a high prevalence of contamination, label conflicts, and activity cliffs (Fig. 4c–e), consistent with our earlier findings and plausibly linked to overlap effects on the protein side.

Examining protein-level properties provides additional context. The primary sequence lengths show a remarkably similar distribution across datasets (Fig. 4f), indicating limited variability in basic structural characteristics. Nearest-neighbour sequence identity (Fig. 4g) is generally high, suggesting strong similarity among proteins within splits; however, the test set displays a broader distribution, pointing to somewhat more diverse sampling at evaluation time. When projecting benchmark proteins into embedding space and comparing them to UniProt (Fig. 4h), we observe that the selected targets cover wide regions of the space, including areas extending beyond the main UniProt distribution.^38^ When restricting the analysis to exact drug–target pairs, the picture changes markedly compared to single-modality leakage statistics. The highest exact pair contamination rate is observed for dataset 3 (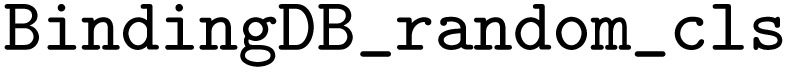), affecting 0.174 % of unique pairs (85 pairs), which remains small in relative terms. For exact pair conflicts under any label discrepancy, dataset 12 (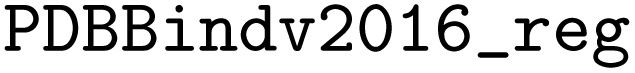) shows the highest relative rate at 0.025 %, although this corresponds to only a single pair, followed closely by 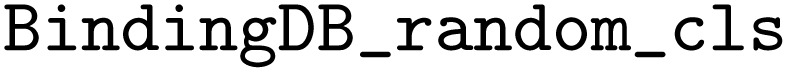 at 0.020 % (10 pairs). Considering task-specific conflict criteria, 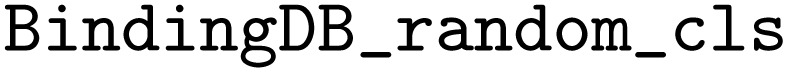 now ranks highest at 0.020 %, whereas 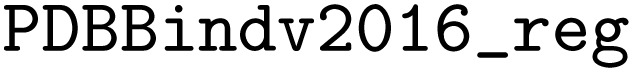 exhibits no severe (≥3σ) pair-level conflicts. In the SI section “Comparison of PDBbind-derived benchmarks,” we looked at Leak Proof PDBbind splits.^18^ In comparison, no exact cross-split drug–target pair contaminations or pair-level conflicts could be observed, consistent with its leak-proof construction. Overall, although many DTI datasets display substantial single-modality leakage on the drug or protein side, exact drug–target pair leakage is comparatively rare, indicating that most structural artefacts arise from modality-specific redundancy rather than duplication of complete interaction pairs.

Despite this apparent coverage, the quantitative evidence shows that models are routinely trained and evaluated on highly similar proteins and ligands across splits, creating a measurable risk that benchmark scores overstate performance on genuinely novel chemistry or target space. This structural proximity, together with documented contamination, conflicts, and redundancy, indicates that some benchmark splits can reward interpolation within familiar molecular or protein neighbourhoods rather than extrapolative abstraction. As a result, reported performance metrics should be interpreted as split- and composition-dependent estimates, especially when claims concern generalisation beyond known ligands, scaffolds, targets, or assays.

### 3.3 Noise degradation experiment

**Fig. 5:**
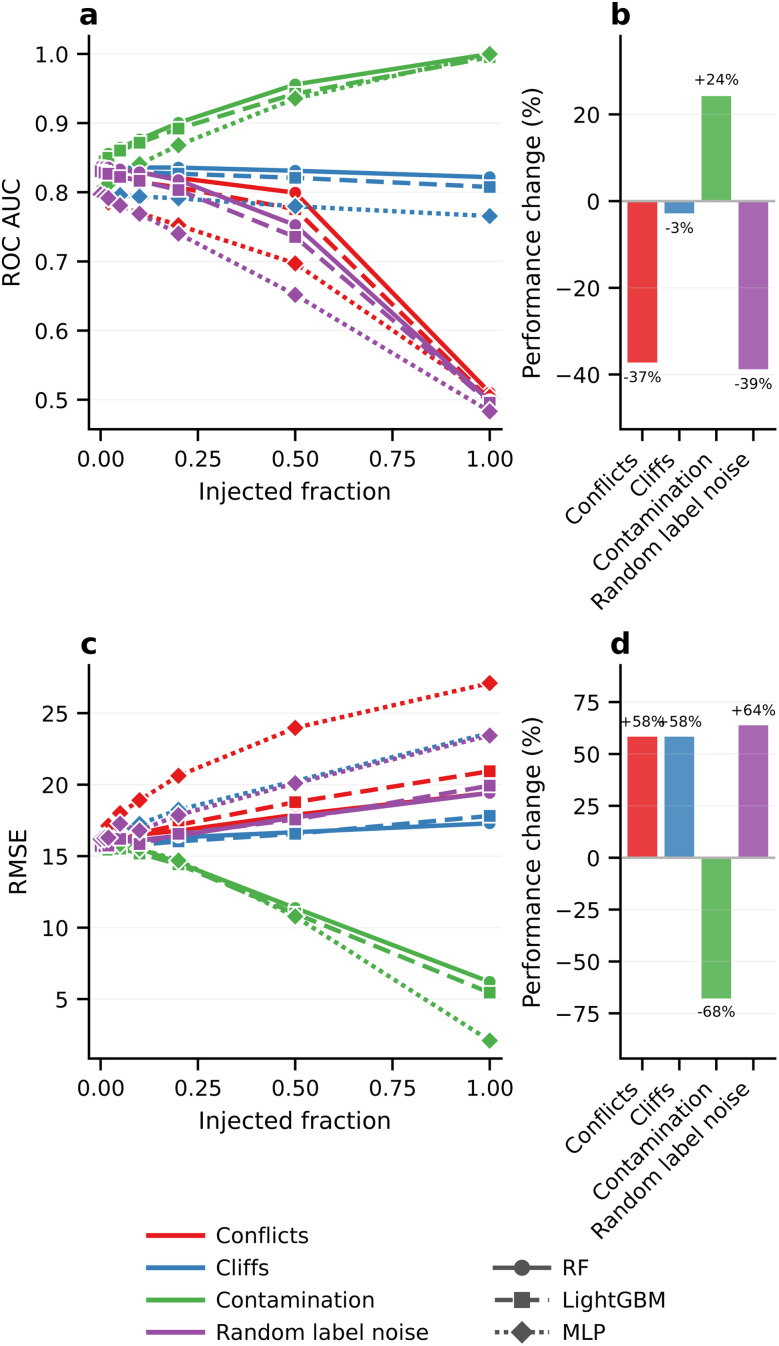
Noise-induced performance degradation across perturbation scenarios. Synthetic perturbations were evaluated on the completed single-task benchmark artefacts with finite train/test labels (41 datasets from DTI, MoleculeNet, and TDC; multitask datasets were skipped). Models were trained on the combined train + validation pool and evaluated on the held-out test split using RF, LightGBM, and a PyTorch MLP. Scenarios include label conflicts, activity cliffs, train–test contamination, and random label noise. Colours denote perturbation scenario; marker shape and line style denote model class. (a) Mean classification ROC AUC across dataset-seed replicates as injected noise fraction increases. (b) Percent change in ROC AUC relative to the fraction-0 baseline for the same dataset, model, scenario, and seed. (c) Mean regression RMSE across dataset-seed replicates. (d) Percent change in RMSE relative to the fraction-0 baseline. For ROC AUC, negative percentage change indicates degradation; for RMSE, positive percentage change indicates degradation. Conflicts and random label noise caused the strongest performance losses, contamination produced apparent performance inflation, and cliffs produced smaller but consistent degradation.

Ultimately, our findings demonstrate that widely used benchmarks suffer from systematic data flaws rather than just inherent task difficulty. We therefore tested whether such flaws can change measured model performance by injecting controlled perturbations into 41 completed single-task datasets with finite train/test labels (27 classification and 14 regression tasks; Fig. 5 and S6). Across RF, LightGBM, and a PyTorch MLP, the perturbations produced directionally consistent effects when each noisy condition was paired with its own fraction-zero baseline. At the highest injected fraction, conflicts and random label noise caused the largest classification losses, with mean ROC AUC decreases of 0.317 and 0.329, respectively, whereas activity-cliff-like perturbations produced a smaller decrease of 0.023. Regression showed the same ordering in RMSE, with mean increases of 6.53 for conflicts, 4.97 for random label noise, and 3.61 for cliffs. By contrast, explicit train–test contamination inflated apparent performance, decreasing paired RMSE by 11.37 and increasing classification performance by 0.177 ROC AUC. These paired effects show that benchmark artefacts can bias measured performance in systematic and statistically significant ways (Fig. 5b–d); for leaderboard interpretation, the key point is that small reported differences may reflect artefact sensitivity rather than robust methodological superiority.^39–42^

### 3.4 Counterfactual benchmark-composition analysis

The noise-injection experiment shows that artefacts can bias model performance when they are deliberately introduced and models are retrained. For leaderboard interpretation, however, a second question is equally important: would the same conclusion hold if the held-out molecules had a different chemical composition? We therefore performed a post-hoc counterfactual evaluation in which all trained-model predictions were kept fixed and only the subset of test molecules used for scoring was resampled. In plain terms, the experiment asks how the leaderboard changes when the evaluation set contains more audit-clean molecules, more molecules that are identical or very similar to training molecules, or more molecules with inconsistent duplicate labels. This simulates alternative but plausible versions of the same benchmark test set.

This analysis was run on 28 completed single-task molecular property datasets with finite held-out labels (16 classification and 12 regression tasks). Across their test rows, 59.2 % were audit-clean, 31.5 % shared a Murcko scaffold with the training set, 7.1 % were near-train analogues, 1.3 % were exact train–test molecular leaks, and 0.9 % had duplicate or conflicting labels. Exact leaks and conflicting labels are true contamination. By contrast, near-train analogues and same-scaffold examples can be scientifically valid local SAR interpolation cases, but they provide weaker evidence for generalisation to new chemical space. Activity cliffs were not treated as contamination. Detailed panel definitions and per-dataset summaries are provided in the SI, “Counterfactual benchmark-composition analysis”.

The original full-test leader was an optimised LightGBM model in 15 of 28 datasets, RF in 7, basic LightGBM in 3, the basic MLP in 2, and linear ECFP in 1. When evaluation was restricted to audit-clean panels, the original leader remained rank 1 in 23 datasets, with a mean rank-1 probability of 0.82. Thus, the leading model was usually not explained simply by exact train–test duplicates or obvious label conflicts. However, the conclusion was less stable when the test panel was enriched for molecules that were identical or highly similar to training molecules: at the observed enrichment level, the original leader's rank-1 probability fell below 0.5 in 15 of 28 datasets, and at 75 % enrichment this occurred in 20 datasets. Matched random panels were also unstable, showing that finite test-panel sampling contributes to this fragility and that analogue-rich examples should not be interpreted as contamination by default.

We used Kendall's *τ* to summarise whether the whole leaderboard was preserved. A value of *τ* = 1 means that the model order is unchanged, whereas lower values indicate more pairwise rank reversals. The mean *τ* relative to the original leaderboard was 0.54 at the observed exact/near-train composition and 0.47 when these examples made up 75 % of the panel. The clearest artefact-specific signal came from label conflicts: conflict-enriched panels were feasible in 14 datasets, reduced the original leader's rank-1 probability below 0.5 in 12 datasets at the observed conflict composition, and lowered the mean *τ* to 0.33. Together, these results support a focused conclusion: benchmark leaderboards are composition-sensitive, and leading-model claims should report whether they remain stable under “clean” datasets.

### 3.5 Data provenance

To understand the root causes of the observed inconsistencies, we also evaluated the data provenance of the analysed benchmarks. These details appear either in the original sources or in consolidated summaries such as MoleculeNet,^25^ TDC,^23,24^ and Polaris.^20,21^ The DTI benchmarks adhere to this pattern. Specifically, BindingDB-derived,^26,43,44^ BioSNAP,^27,43,44^ Human DTI,^28,43–45^ and PDBbind-derived^29–32^ tasks specify ligand–target pairs, interaction or affinity labels, and split semantics. These tasks are traceable to curated interaction databases or structural resources with documented assay contexts. Reproducibility is supported by open-source loaders and standardised splitting utilities. DeepChem's 
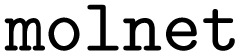
 module, the TDC Python interface, and Polaris's GitHub resources provide deterministic implementations of random, scaffold, and challenge-preserved splits.^20,21,23,25^ These DTI datasets have the following configurations: random, scaffold-, cluster-, and protein-based splits. These widely used splits originate from a small number of publications and are typically distributed as fixed files. While this reduces overt test-set leakage, it has produced multiple, slightly incompatible variants of nominally identical benchmarks and still permits subtle overfitting to public test labels.

However, trusted provenance and reproducible splits do not guarantee data quality. Established benchmarks may still exhibit structural inconsistencies, ambiguous labels, heterogeneous assay conditions, and substantial redundancy, especially in DTI settings where ligands and protein sequences recur across splits. These issues make systematic auditing essential: transparent lineage must be complemented by quantitative checks. This ensures that benchmark-driven conclusions reflect genuine methodological progress rather than artefacts of the underlying data.

## 4 Conclusions and outlook

Transparent data provenance and reproducible splits are foundational to reliable ML, yet our systematic audit demonstrates that they are not sufficient to ensure data quality. Across widely used benchmark suites, including MoleculeNet, TDC, Polaris, and multiple DTI datasets, we identified pervasive structural weaknesses. These include substantial cross-split leakage and unresolved label conflicts, all of which distort the effective difficulty of the tasks. In addition, several datasets contain compounds of questionable medicinal-chemical plausibility, reflected by frequent REOS warnings. Within DTI benchmarks in particular, the apparent abundance of interaction data conceals a heavy dependence on a comparatively small set of unique protein targets, leading to extensive target-side overlap and strikingly high sequence similarity between training and test splits.

Benchmark leaderboards are often divided by marginal performance differences, so these vulnerabilities have an outsized influence on the field. High-capacity models may exploit leakage patterns or structural redundancies, but the broader risk is conclusion fragility: leading-model rankings can depend on whether test panels are audit-clean, conflict-rich, analogue-rich, or randomly resampled at matched size. When evaluated under synthetic perturbations or counterfactual benchmark compositions, headline metrics and relative rankings can shift substantially. Importantly, this does not imply that near-train analogues or same-scaffold examples are erroneous, nor that a leading architecture is intrinsically invalid. Rather, it shows that benchmark claims should state the evaluation composition under which they hold. This pattern suggests that a non-trivial fraction of leading-model claims reflects sensitivity to evaluation artefacts and panel composition rather than robust methodological superiority.

These shortcomings can be identified by pairing transparent sourcing and curation with rigorous, quantitative auditing. Our work contributes through concrete steps towards this goal. We provide a flexible, YAML-driven auditing toolkit for biomolecular benchmarks that integrates seamlessly into existing workflows. Furthermore, we offer actionable guidance for benchmark construction, outlining curation strategies aligned with specific scientific objectives instead of leaderboard optimisation.

Looking ahead, emerging initiatives such as OpenADMET^46^ or Polaris^20^ illustrate a broader shift towards higher standards in dataset engineering, combining stable interfaces with industry-grade curation practices. By prioritising systematic measurement, continuous auditing, and reproducible evaluation, our framework equips researchers, dataset curators, and reviewers with the tools needed to identify robust tasks, diagnose structural risks, and conduct comparisons that are far more likely to translate into meaningful real-world impact beyond leaderboard performance.

## 5 Methods

### 5.1 Pipeline overview

To perform these audits reproducibly, we implemented a unified pipeline that accepts dataset configurations and produces standardised diagnostic outputs. A single entry point script reads YAML configuration files from disk, constructs the appropriate data loader and analyser objects, and writes analysis artefacts into a dedicated output directory. The same infrastructure serves small-molecule property tasks (“SMILES-only”) and DTI tasks (“paired SMILES and amino-acid sequences”), enabling a unified treatment of contamination, similarity, and label consistency across heterogeneous sources.

For each benchmark, the pipeline produces a set of standardised artefacts: a summary of high-level statistics (split sizes, hygiene, and similarity structure); a record-level table preserving the analyser's internal view; and detailed line-oriented files documenting specific conflict and cliff events. For DTI tasks, representative sequence alignments are also generated.

### 5.2 Configurability

The configurability of the pipeline is centred around the YAML configuration files. Each configuration declares a “type” or “modality” that uniquely determines the associated loader and analyser classes. When 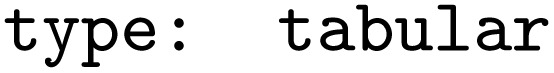 is specified, the loader ingests CSV, TSV, or Parquet files and infers column roles based on explicit instructions provided in the 
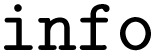
 block.

The SMILES column can be set explicitly through 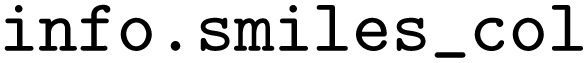 or automatically resolved from a curated list of common column names. Label information is extracted from either a single column (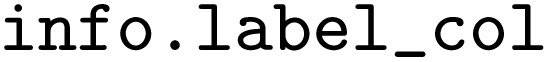) or multiple columns (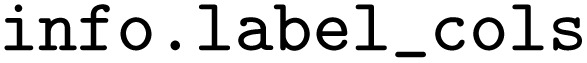), with multi-column labels stored as vectors to preserve structural correspondence. Additional optional fields, including molecular identifiers (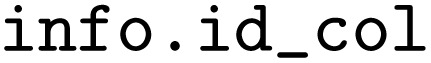), amino-acid sequences (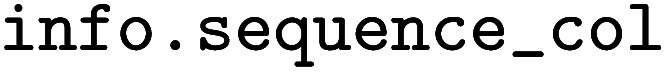), and target identifiers (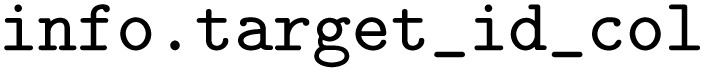), are propagated without transformation. For DTI configurations (specified by 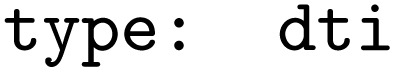 or 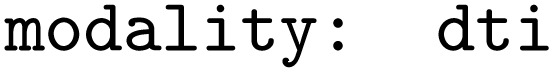), the loader enforces the presence of an amino-acid sequence column and does not drop invalid SMILES unless 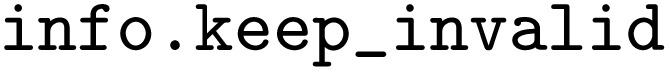 is set to false, thereby ensuring ligand–target alignment.

Structure preprocessing is controlled through the cleaning configuration. By default, each SMILES string is processed by an RDKit-based 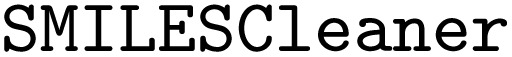 that canonicalises structures, standardises tautomers, removes duplicates, and annotates REOS alerts. Users may disable cleaning or replace the cleaner by providing a fully qualified Python import path in 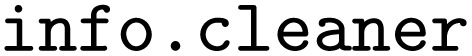. The flag 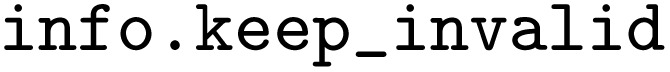 dictates whether rows that fail cleaning are retained (with explicit invalid markers) or removed. Retention is often necessary for DTI datasets, where dropping rows would disrupt ligand–target pairing.

Similarity and activity cliff detection rely on circular Morgan/ECFP fingerprints computed with a configurable radius (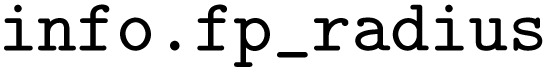, default 2) and bit length (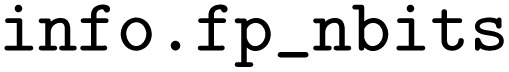, default 2048). A unified similarity threshold (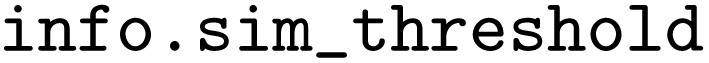, default 0.9) is applied across three modalities: molecular fingerprint Tanimoto similarity, scaffold fingerprint similarity based on generic Murcko scaffolds, and normalised Levenshtein similarity of SMILES. Pairs meeting or exceeding any of these criteria are classified as similar. These definitions drive conflict detection, activity cliff identification, and near-duplicate analyses throughout the pipeline.

Extensibility is achieved through modular factories defined in 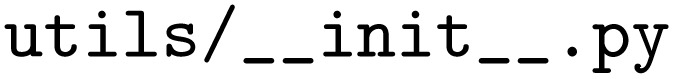. New benchmarks can be incorporated by adding their data files to the 
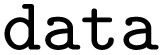
/ directory and defining a corresponding YAML configuration that specifies the type, task, and column semantics. Custom loader or analyser classes can be integrated with minimal modifications to the factory logic. This modular design enables consistent application of the analytical framework across existing and newly introduced datasets.

### 5.3 Cleaner

The cleaning workflow applies a compact, deterministic sequence of steps to each input SMILES string. First, structures are canonicalised by converting SMILES to an RDKit molecule and generating canonical SMILES and InChI identifiers; failures mark the entry as invalid. Second, duplicates are removed by comparing canonical SMILES and InChI, retaining only the first occurrence. Third, molecules are standardised through RDKit's cleanup, parent-fragment selection, charge neutralisation, and tautomer canonicalisation, followed by external molblock normalisation; unsuccessful transformations result in invalidation. Fourth, successfully standardised structures receive a quality score from a rule-based structural checker. Finally, REOS medicinal-chemistry filters annotate any rule violations and count the number of failed checks. The result is a canonical, deduplicated, standardised set of SMILES strings with associated InChI identifiers and diagnostic metadata.

### 5.4 Audit metrics

For each run, we define the reference split tv as the concatenation of train and validation when validation is present, and as train-only otherwise. All ligand-side metrics operate on cleaned SMILES strings (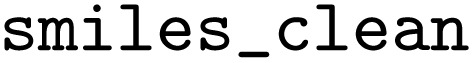) produced by the loader.

#### 5.4.1 Hygiene and contamination

We quantify duplicate burden within and across splits by counting repeated 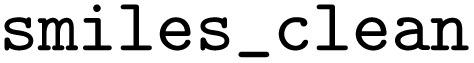 values. To measure cross-split contamination, we compute the size of the overlap between the unique cleaned SMILES sets in the train, validation and test sets, |*S*_tv_ ∩ *S*_test_|. We also summarise chemical-filter warnings by computing the mean and standard deviation of the REOS warning count in tv when available.^36^

#### 5.4.2 Nearest-neighbour similarity

Nearest-neighbour similarity is computed using Morgan/ECFP fingerprints and Tanimoto similarity. For each ligand in a query split (validation or test), we compute its maximum Tanimoto similarity to any ligand in a reference split (train or train + valid) and report the mean and standard deviation of these nearest-neighbour similarities (default: 50).^33^

#### 5.4.3 Label conflicts

Conflicts are defined on identical cleaned SMILES strings with divergent labels. For classification tasks, a conflict occurs when at least two rows with the same 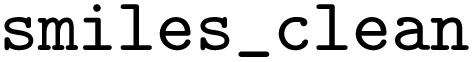 have different labels. For regression tasks, we compute the standard deviation *σ*_tv_ of the tv labels and flag a conflict when any within-group label difference exceeds the threshold of 3*σ*_tv_.

#### 5.4.4 Activity cliffs

Activity cliffs are defined on pairs of distinct ligands that are considered “similar” but have divergent labels.^13^ Similarity uses a consensus rule with threshold *τ* (default *τ* = 0.9): a pair is similar if either (i) ECFP Tanimoto similarity ≥*τ*, (ii) generic Murcko-scaffold ECFP Tanimoto similarity ≥*τ*, or (iii) normalised SMILES string similarity ≥*τ* (Levenshtein ratio when available, with a deterministic fallback).^13^ For classification, a cliff is a similar pair with mismatched labels; for regression, a cliff is a similar pair with |Δ*y*| ≥ 3*σ*_tv_.

### 5.5 DTI target leakage diagnostics

For DTI benchmarks, the pipeline applies the ligand-side audit described above and adds target-side diagnostics based on normalised amino-acid sequences. Target sequences are normalised to uppercase with whitespace removed prior to overlap and similarity computations.

#### 5.5.1 Sequence hygiene and overlap

We compute the number of unique target sequences per split, cross-split overlaps, and repeated sequences within splits. In addition, we report two DTI-specific cross-split conflict types: identical (ligand and target) pairs appearing in more than one split and target sequences that appear across splits and are associated with multiple unique ligands.

#### 5.5.2 Sequence similarity

Nearest-neighbour target-sequence similarity is quantified using global pairwise alignment *via* the EMBOSS^34^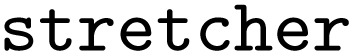 program. For each query sequence, the analyser selects the reference sequence with maximal identity (ties are broken by alignment score), summarises the distribution of nearest-neighbour identity values, and records representative high-identity alignments for inspection.

### 5.6 Noise degradation protocol

To quantify the sensitivity of model performance to dataset issues, we implemented a controlled perturbation study on all single-task benchmark datasets. Multitask datasets were excluded so that perturbations and metrics were defined for a single label per row; this excluded MoleculeNet ClinTox, SIDER, and Tox21. The experiment attempted 48 single-task artefacts.

For each completed dataset, we used the split assignments stored in the run artefacts. The model training pool was the union of the training and validation splits, and evaluation was performed only on the held-out test split. Noise was injected at fractions of 0, 0.01, 0.02, 0.05, 0.1, 0.2, 0.5, and 1.0, using three random seeds. For conflicts, cliffs, and random label noise, the fraction was defined relative to the number of training-pool rows. For contamination, the fraction was defined relative to the number of test rows copied into the training pool.

We evaluated four perturbation scenarios. In the “conflicts” scenario, selected training rows were duplicated with identical inputs but contradictory labels. Binary classification labels were flipped, while regression labels were shifted by approximately three training-label standard deviations with random sign. In the “cliffs” scenario, selected training labels were modified using high-similarity neighbours. Ligand similarity was computed from Morgan fingerprints and a Tanimoto threshold of 0.9; up to 256 candidate neighbours were considered per selected row. For DTI datasets, cliff pairs additionally required the same target sequence. Classification labels were forced opposite to the selected neighbour, and regression labels were replaced by the neighbour label plus or minus an approximately three-standard-deviation shift. Rows without an eligible neighbour were left unchanged. In the “contamination” scenario, selected test rows and their labels were copied into the training pool, creating explicit train–test overlap. In the “random label noise” scenario, selected training labels were reassigned by sampling from the empirical finite training-label distribution.

We compared three model classes. RF classifiers and regressors were implemented with scikit-learn using 300 trees.^47^ LightGBM classifiers and regressors used 400 boosting rounds and a learning rate of 0.05.^48^ The neural baseline was a minimal PyTorch/Lightning MLP.^49^ Molecular inputs were represented with Morgan fingerprint features, and DTI inputs additionally included hashed target-sequence features. The MLP used a sparse-safe standardisation step, one hidden layer with 100 ReLU units, and one scalar output. Binary classification used binary cross-entropy with logits during training and sigmoid probabilities for evaluation; regression used mean squared error loss. Optimisation used Adam with a learning rate of 10^−3^, a weight decay of 10^−4^, a batch size of min(200, *n*_train_), and a maximum of 200 epochs.

For classification, probability outputs were used to compute ROC AUC, precision recall area under the curve (PR AUC), balanced accuracy, accuracy, F1, precision, recall, Matthews correlation coefficient, log loss, Brier score, evaluation-set size, and test positive rate. Threshold-dependent metrics used a threshold of 0.5. For regression, we computed mean squared error (MSE), RMSE, mean absolute error (MAE), *R*^2^, explained variance, Pearson *r*, Spearman *ρ*, Kendall *τ*, maximum error, and evaluation-set size.

For statistical analysis, each noisy condition was paired with its own fraction-zero baseline for the same dataset, model, scenario, and seed. For ROC AUC and PR AUC, degradation was defined as baseline minus noisy performance. For RMSE and MAE, degradation was defined as noisy error minus baseline error. Thus, positive values consistently indicate worse performance, whereas negative values indicate apparent performance improvement. Conflicts, cliffs, and random label noise were tested with one-sided Wilcoxon signed-rank tests^50^ for degradation greater than zero. Contamination was tested in the opposite direction because leakage is expected to inflate apparent performance. Grouped tests were corrected using Benjamini–Hochberg false discovery rate (FDR) control.^51^ The behaviour was summarised using the Spearman correlation between injected fraction and expected effect magnitude, with the contamination sign flipped before trend estimation.

### 5.7 Counterfactual benchmark-composition protocol

We implemented the counterfactual analysis as a post-hoc rank-fragility protocol for single-task molecular property datasets with finite test labels. For each dataset, trained-model prediction files were generated from the fixed original split using six fingerprint-based model variants: linear ECFP, RF, basic and optimised LightGBM, and basic and optimised PyTorch MLP. No model was retrained during counterfactual evaluation; only the test molecules used for scoring were resampled.

Test rows were standardised and annotated for exact train–test molecular identity leakage, duplicate or conflicting labels, maximum ECFP/Tanimoto similarity to the training set, near-train analogue status at a primary threshold of 0.85, and same Murcko-scaffold overlap with the training set. Invalid molecules were retained in audit counts but excluded from fingerprint-similarity calculations. Activity cliffs were intentionally not used as contamination flags.

Counterfactual panels represented audit-clean evaluation, the observed test-set composition, enrichment for exact or near-training-set molecules, enrichment for label-conflicting molecules, and matched random controls. Panels were sampled without duplicate molecules within a panel and were stratified by class label or regression-label quantile where feasible. For each feasible panel composition, we recomputed model metrics, ranks, rank-1 probabilities, leading-model-versus-RF margins, and Kendall rank correlation to the original full-test leaderboard. Detailed target rates, panel-size rules, and infeasibility handling are provided in the SI.

### 5.8 Output

For each of the 51 configurations, the audit writes a per-dataset run directory containing at least 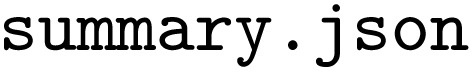 and 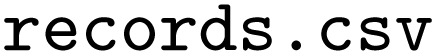. Drill-down artefacts are emitted when non-empty: 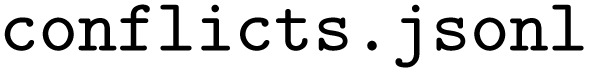 was produced for 27 datasets and 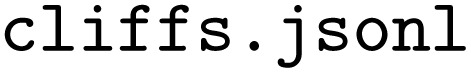 for 46 datasets. For DTI benchmarks, target leakage diagnostics produced 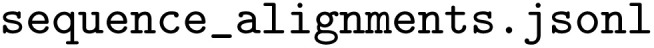 for all 13 runs, and 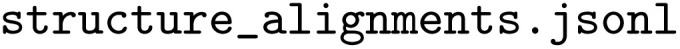 when Foldseek^52^ inputs were available.

### 5.9 Runtime

Runtime scales quadratically with the evaluated split sizes. For molecular property benchmarks, the dominant term is the number of ligand-pair comparisons,
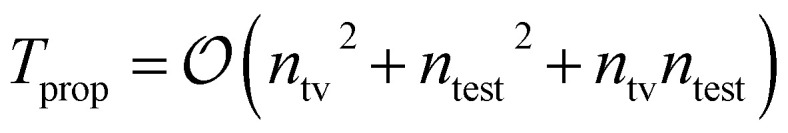
where *n*_tv_ = *n*_train_ + *n*_valid_.

For DTI benchmarks, the same ligand-side term is retained, but runtime is dominated by target-sequence alignment:
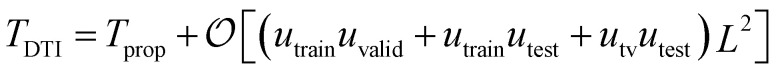
where *u* denotes unique target sequences, *u*_tv_ = |*U*_train_ ∪ *U*_valid_|, and *L* is the average sequence length.

## Author contributions

Conceptualisation: M. G. S.; data curation: M. G. S.; formal analysis: M. G. S. and A. D.; investigation: M. G. S. and A. D.; methodology: M. G. S. and A. D.; software: M. G. S. and A. D.; supervision: S. A. S.; writing – original draft: M. G. S. and A. D.; writing – review and editing: all authors. All authors have given approval to the final version of the manuscript.

## Conflicts of interest

There are no conflicts to declare.

## Supplementary Material

SC-OLF-D6SC01799A-s001

## Data Availability

The auditing pipeline can be installed *via* pip install benchaudit. Comprehensive documentation, along with the source code and data, is openly available at https://github.com/sieber-lab/benchaudit and https://sieber-lab.github.io/benchaudit. The system used for computational work is equipped with an AMD Ryzen Threadripper PRO 5995WX CPU with 64/128 cores/threads and 1024 GB RAM. The server is also powered by an NVIDIA RTX 4090 GPU with 24 GB VRAM. Supplementary information (SI): additional results and discussion. See DOI: https://doi.org/10.1039/d6sc01799a.

## References

[cit1] Schaefer H. F. (1986). Science.

[cit2] Schlick T., Collepardo-Guevara R., Halvorsen L. A., Jung S., Xiao X. (2011). Q. Rev. Biophys..

[cit3] Schlick T., Portillo-Ledesma S. (2021). Nat. Comput. Sci..

[cit4] Chen H., Engkvist O., Wang Y., Olivecrona M., Blaschke T. (2018). Drug Discovery Today.

[cit5] Baskin I. I., Winkler D., Tetko I. V. (2016). Expert Opin. Drug Discovery.

[cit6] de Almeida A. F., Moreira R., Rodrigues T. (2019). Nat. Rev. Chem..

[cit7] Mohammed S., Budach L., Feuerpfeil M., Ihde N., Nathansen A., Noack N., Patzlaff H., Naumann F., Harmouch H. (2025). Inf. Syst..

[cit8] Jain A. , Patel H., Nagalapatti L., Gupta N., Mehta S., Guttula S., Mujumdar S., Afzal S., Sharma Mittal R. and Munigala V., Proceedings of the 26th ACM SIGKDD International Conference on Knowledge Discovery & Data Mining, New York, NY, USA, 2020, pp. 3561–3562

[cit9] Priestley M., O’donnell F., Simperl E. (2023). J. Data Inf. Qual..

[cit10] Fourches D., Muratov E., Tropsha A. (2010). J. Chem. Inf. Model..

[cit11] Fourches D., Muratov E., Tropsha A. (2016). J. Chem. Inf. Model..

[cit12] Helma C., Kramer S., Pfahringer B., Gottmann E. (2000). Environ. Health Perspect..

[cit13] van Tilborg D., Alenicheva A., Grisoni F. (2022). J. Chem. Inf. Model..

[cit14] Eriksson M. , Purificato E., Noroozian A., Vinagre J., Chaslot G., Gomez E. and Fernandez-Llorca D., Proceedings of the AAAI/ACM Conference on AI, Ethics, and Society, 2025, vol. 8, pp. 850–864

[cit15] Buttenschoen M., Morris G. M., Deane C. M. (2024). Chem. Sci..

[cit16] Kramer C., Chodera J., Damm-Ganamet K. L., Gilson M. K., Günther J., Lessel U., Lewis R. A., Mobley D., Nittinger E., Pecina A., Schapira M., Walters W. P. (2025). J. Chem. Inf. Model..

[cit17] Wang Y., Sun K., Li J., Guan X., Zhang O., Bagni D., Zhang Y., Carlson H. A., Head-Gordon T. (2025). Digital Discovery.

[cit18] Li J., Guan X., Zhang O., Sun K., Wang Y., Bagni D., Head-Gordon T. (2026). J. Phys. Chem. B..

[cit19] Graber D., Stockinger P., Meyer F., Mishra S., Horn C., Buller R. (2025). Nat. Mach. Intell..

[cit20] Wognum C. , Zhu L., Mary H., St-Laurent J., Larissa, Quirke A., Hounwanou H., Roselyn, Zhu K., Whitfield S., Burns J., Howe K., Howe (McLean) K. and Felix, Polaris-Hub/Polaris: 0.12.1, Zenodo, 2025, https://zenodo.org/records/15610218

[cit21] Fang C., Wang Y., Grater R., Kapadnis S., Black C., Trapa P., Sciabola S. (2023). J. Chem. Inf. Model..

[cit22] Fluetsch A., Trunzer M., Gerebtzoff G., Rodríguez-Pérez R. (2024). Chem. Res. Toxicol..

[cit23] Huang K. , Fu T., Gao W., Zhao Y., Roohani Y., Leskovec J., Coley C. W., Xiao C., Sun J. and Zitnik M., Therapeutics Data Commons: Machine Learning Datasets and Tasks for Drug Discovery and Development, 2021

[cit24] Huang K., Fu T., Gao W., Zhao Y., Roohani Y., Leskovec J., Coley C. W., Xiao C., Sun J., Zitnik M. (2022). Nat. Chem. Biol..

[cit25] Wu Z., Ramsundar B., Feinberg E. N., Gomes J., Geniesse C., Pappu A. S., Leswing K., Pande V. (2018). Chem. Sci..

[cit26] Liu T., Lin Y., Wen X., Jorissen R. N., Gilson M. K. (2007). Nucleic Acids Res..

[cit27] Zitnik M. , Sosič R., Maheshwari S. and Leskovec J., BioSNAP Datasets: Stanford Biomedical Network Dataset Collection, http://snap.stanford.edu/biodata, 2018

[cit28] Huang K., Xiao C., Glass L. M., Sun J. (2021). Bioinformatics.

[cit29] Li S. , Zhou J., Xu T., Huang L., Wang F., Xiong H., Huang W., Dou D. and Xiong H., Proceedings of the 27th ACM SIGKDD Conference on Knowledge Discovery & Data Mining, New York, NY, USA, 2021, pp. 975–985

[cit30] Su M., Yang Q., Du Y., Feng G., Liu Z., Li Y., Wang R. (2019). J. Chem. Inf. Model..

[cit31] Lutag W. , Wutag Q., Zhangtag J., Raotag J., Lixtag C. and Zheng S., TANKBind: Trigonometry-Aware Neural NetworKs for Drug-Protein Binding Structure Predictiontag, 36th Conference on Neural Information Processing Systems (NeurIPS 2022), 2022

[cit32] Stärk H. , Ganea O., Pattanaik L., Barzilay D. R. and Jaakkola T., Proceedings of the 39th International Conference on Machine Learning, 2022, pp. 20503–20521

[cit33] Landrum G. , Tosco P., Kelley B., Sriniker, Gedeck, Schneider N., Vianello R., Ric, Dalke A., Cole B., Savelyev A., Swain M., Turk S., D. N., Vaucher A., Kawashima E., Wójcikowski M., Probst D., Godin G., Cosgrove D., Pahl A., J. P., Berenger F., Strets, Varjo J. L., O'Boyle N., Fuller P., Jensen J. H., Sforna G. and Gavid D., Rdkit/Rdkit: 2020_03_1 (Q1 2020) Release, Zenodo, 2020

[cit34] Rice P., Longden I., Bleasby A. (2000). Trends Genet..

[cit35] Mendez D., Gaulton A., Bento A. P., Chambers J., De Veij M., Félix E., Magariños M. P., Mosquera J. F., Mutowo P., Nowotka M., Gordillo-Marañón M., Hunter F., Junco L., Mugumbate G., Rodriguez-Lopez M., Atkinson F., Bosc N., Radoux C. J., Segura-Cabrera A., Hersey A., Leach A. R. (2019). Nucleic Acids Res..

[cit36] Walters W. P., Murcko A. A., Murcko M. A. (1999). Curr. Opin. Chem. Biol..

[cit37] Hayes T., Rao R., Akin H., Sofroniew N. J., Oktay D., Lin Z., Verkuil R., Tran V. Q., Deaton J., Wiggert M., Badkundri R., Shafkat I., Gong J., Derry A., Molina R. S., Thomas N., Khan Y. A., Mishra C., Kim C., Bartie L. J., Nemeth M., Hsu P. D., Sercu T., Candido S., Rives A. (2025). Science.

[cit38] The UniProt Consortium (2025). Nucleic Acids Res..

[cit39] Jia R. and Liang P., Proceedings of the 2017 Conference on Empirical Methods in Natural Language Processing, Copenhagen, Denmark, 2017, pp. 2021–2031

[cit40] Reimers N. and Gurevych I., Proceedings of the 2017 Conference on Empirical Methods in Natural Language Processing, Copenhagen, Denmark, 2017, pp. 338–348

[cit41] Hendrycks D. and Dietterich T., Benchmarking Neural Network Robustness to Common Corruptions and Perturbations, 2019

[cit42] Henderson P. , Islam R., Bachman P., Pineau J., Precup D. and Meger D., Proceedings of the Thirty-Second AAAI Conference on Artificial Intelligence and Thirtieth Innovative Applications of Artificial Intelligence Conference and Eighth AAAI Symposium on Educational Advances in Artificial Intelligence, New Orleans, Louisiana, USA, 2018, pp. 3207–3214

[cit43] Kang H., Goo S., Lee H., Chae J.-w., Yun H.-y., Jung S. (2022). Pharmaceutics.

[cit44] Koh H. Y., Nguyen A. T. N., Pan S., May L. T., Webb G. I. (2024). Nat. Mach. Intell..

[cit45] Davis M. I., Hunt J. P., Herrgard S., Ciceri P., Wodicka L. M., Pallares G., Hocker M., Treiber D. K., Zarrinkar P. P. (2011). Nat. Biotechnol..

[cit46] MacDermott-Opeskin H., Scheen J., Wognum C., Horton J. T., West D., Payne A. M., Castellanos M. A., Colby S., Griffen E., Cousins D., Stacey J., Reid L., Aschenbrenner J. C., Fearon D., Balcomb B., Marples P., Tomlinson C. W. E., Lithgo R., Godoy A. S., Winokan M., Barr H., Lahav N., Lavi M., Duberstein S., Cohen G., Fate G., Lefker B., Robinson R., Szommer T., Lynch N., Minh D. D. L., Thuy La V. N., Kang L., Huddleston K., Renslow R., Tollefson M., Walters W. P., Xu C., Hsu J., St-Laurent J., Etsmoberg H., Zhu L., Quirke A., Haleem M. I. A., Alibay I., Baid G., Birnbaum B., Bishop K. P., Bohorquez H., Bose A., Brown C. J., Burns J., Cai L., Cedeno R., de Cesco S., Chupakhin V., Clark F., Cole D. J., Corbi-Verge C., Danial M., Davi A., Dehaen W., Piet Doering N., Dougha A., Dréanic M.-P., Eakin B., Ehrlich A., Elijosius R., Fülöp J., Gitter A., Goossens K., Gu Y., Head-Gordon T., Hoffer L., Hofmans J., Jiang E., Kaminow B., Khosravi S., Khoualdi A. F., Lenselink E. B., Liu Z., Liu Y., Liu S., Ma Y., Maher P., Mayer I., Mendez-Lucio O., Mey A. S. J. S., Michel J., Montanari F., Niu T., Ogino R., Palaniappan A., Pan X., Patnaik A., Pham L.-H., Pinto L., Purnomo J., Rich A., Schaaf L., Schran C., Singh R. K., Srilakshmi M., Srivastava S. P., Sun K., Sun Z., Talagayev V., Balakrishnan B. T. S., Titus I., Tkatchenko A., Treyde W., Tricarico G., Tripp A., Vithayapalert N., Wang Y., Wasi A. T., Wedig S., Wolber G., Xu B., Zhou W., Delft F. v., Lee A., Kirkegaard K., Sjö P., Fraser J. S., Chodera J. D. (2026). J. Chem. Inf. Model..

[cit47] Pedregosa F., Varoquaux G., Gramfort A., Michel V., Thirion B., Grisel O., Blondel M., Prettenhofer P., Weiss R., Dubourg V., Vanderplas J., Passos A., Cournapeau D., Brucher M., Perrot M., Duchesnay É. (2011). J. Mach. Learn. Res..

[cit48] Ke G. , Meng Q., Finley T., Wang T., Chen W., Ma W., Ye Q. and Liu T.-Y., Advances in Neural Information Processing Systems, 2017

[cit49] Paszke A. , Gross S., Massa F., Lerer A., Bradbury J., Chanan G., Killeen T., Lin Z., Gimelshein N., Antiga L., Desmaison A., Kopf A., Yang E., DeVito Z., Raison M., Tejani A., Chilamkurthy S., Steiner B., Fang L., Bai J. and Chintala S., Advances in Neural Information Processing Systems, 2019

[cit50] Wilcoxon F. (1945). Biom. Bull..

[cit51] Benjamini Y., Hochberg Y. (1995). J. R. Stat. Soc. Ser. B Methodol..

[cit52] van Kempen M., Kim S. S., Tumescheit C. (2024). Fast and accurate protein structure search with Foldseek. Nat. Biotechnol..

