# Supplementary material for: Auditing widely used biomolecular benchmarks reveals systematic data inconsistencies
Source: Chem Sci. 2026 Jul 22;17(36):17455–67. doi: 10.1039/d6sc01799a (PMC13425739; doi:10.1039/d6sc01799a)
Supplement: Supplementary file 1 [file SC-017-D6SC01799A-s001.pdf]

Supplementary Information (SI) for Chemical Science.
This journal is © The Royal Society of Chemistry 2026

SI: Auditing widely used biomolecular benchmarks reveals systematic data inconsistencies

Additional results and discussion

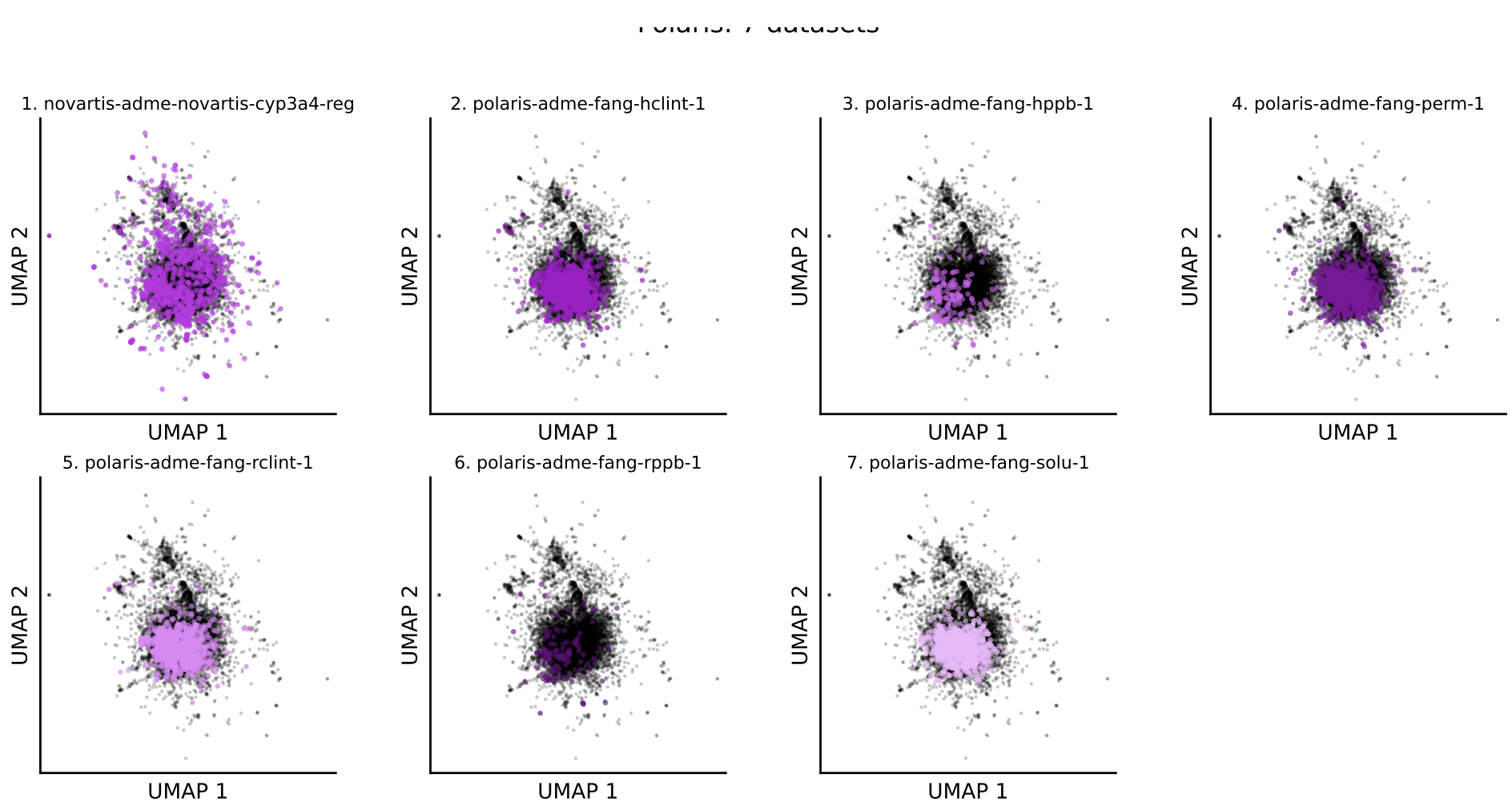

Fig. S1: Chemical space of Polaris per benchmark. Comparison to 10 000 random samples from ChEMBL36.[1]

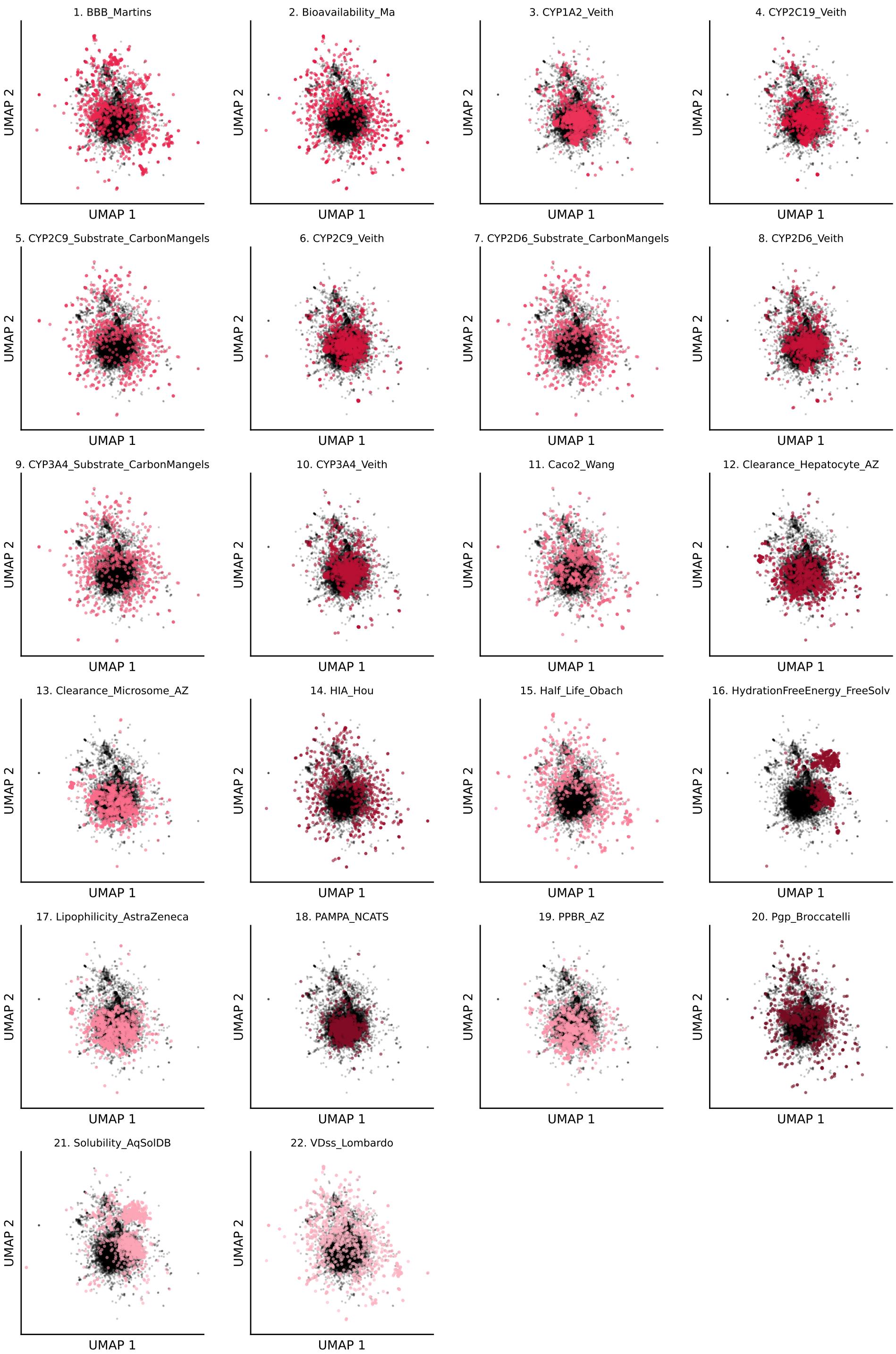

Fig. S2: Chemical space of TDC per benchmark. Comparison to 10 000 random samples from ChEMBL36.[1]

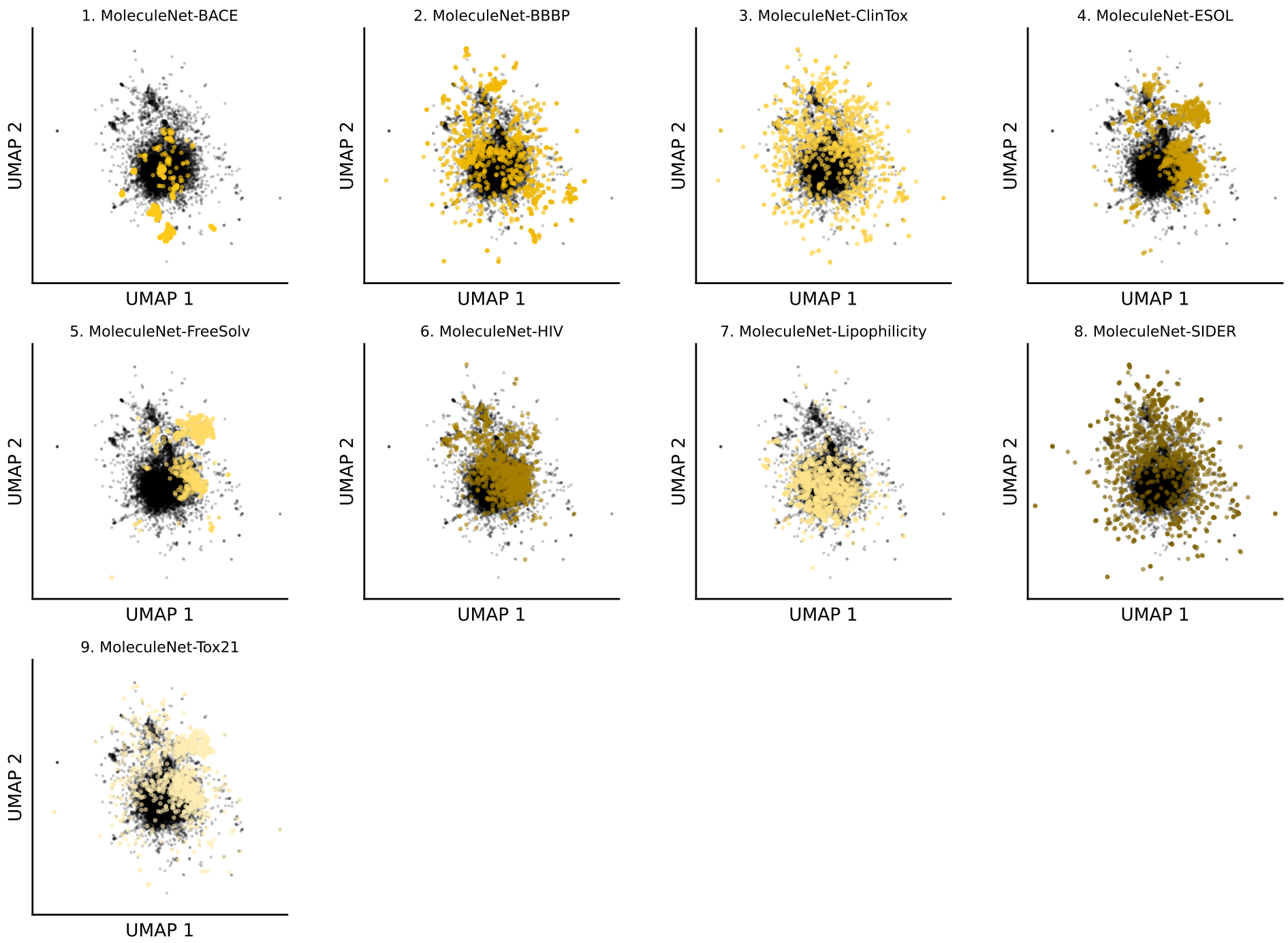

Fig. S3: Chemical space of MoleculeNet per benchmark. Comparison to 10 000 random samples from ChEMBL36.[1]

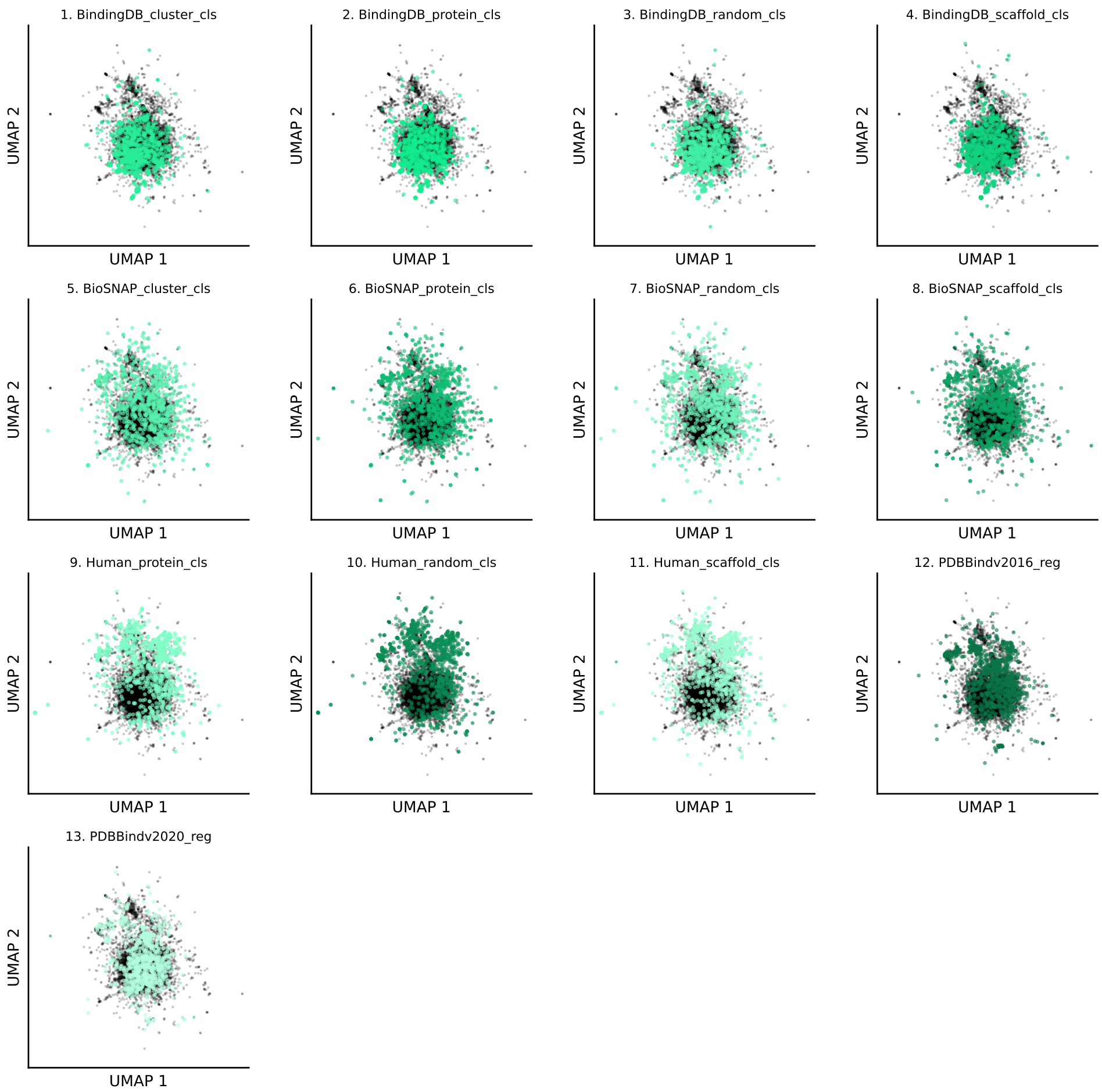

Fig. S4: Chemical space of DTI per benchmark. Comparison to 10 000 random samples from ChEMBL36.[1]

Comparison of PDBBind-derived benchmarks

We compared PDBBind v2016, PDBBind v2020, and the added LP-PDBBind CL1[2] non-covalent benchmark using the same BenchAudit metrics (Tab. S1): exact ligand contamination, exact target contamination, cross-split label conflicts, exact cross-split DTI pair conflicts, sequence-level multi-ligand conflicts, and train/validation–test activity cliffs. The conventional PDBBind splits show substantial exact overlap between train/validation and test. PDBBind v2016 has 56 overlapping unique test ligands (19.4%) and 81 overlapping unique test targets (36.7%), while PDBBind v2020 has 48 overlapping unique test ligands (14.0%) and 39 overlapping unique test targets (14.1%). In contrast, LP-PDBBind CL1 has a much larger test set but lower relative exact overlap: 154 overlapping unique test ligands (4.4%) and 60 overlapping unique test targets (2.6%).

Tab. S1: Consistent BenchAudit comparison of the three PDBBind-derived DTI regression benchmarks. Ligand and target contamination report exact train/validation–test overlap, with percentages computed over unique test ligands or unique test targets. Label conflicts are severe train/validation–test regression conflicts for identical ligands. DTI pair conflicts are exact ligand–target pair recurrences across splits. Cliffs are train/validation–test activity-cliff pairs at the same similarity threshold used throughout BenchAudit.

Dataset	Lig. cont.	Target cont.	Label confl.	DTI confl.	Seq.–ligand confl.	Cliffs
PDBBind v2016	56 (19.4%)	81 (36.7%)	0	2	178	8
PDBBind v2020	48 (14.0%)	39 (14.1%)	2	4	368	37
LP-PDBBind	154 (4.4%)	60 (2.6%)	1	0	73	117

Conflict metrics show the same trend. Exact cross-split DTI pair conflicts are present in PDBBind v2016 and v2020 (2 and 4, respectively) but are eliminated in LP-PDBBind CL1. Sequence-level multi-ligand conflicts also decrease from 178 and 368 in the conventional splits to 73 in LP-PDBBind CL1. Cross-split activity cliffs remain in LP-PDBBind, with 117 detected pairs, reflecting that the split is not free of local structure–activity relationships. However, the exact contamination and exact DTI-pair leakage metrics are markedly reduced, supporting the intended role of LP-PDBBind as a cleaner generalisation-oriented processed form. Overall, BenchAudit showcases the known limitations of the conventional PDBBind splits and, using the same automated criteria, confirms that the LP-PDBBind split substantially improves exact ligand and target split hygiene.

Manual inspection of activity cliffs

To qualitatively compare activity-cliff behaviour in datasets with contrasting audit quality, we selected the cleanest and less cleanest datasets according to the held-out test-set non-clean fraction from the dataset audit. The cleanest dataset was MoleculeNet-BBBP ($f_{\text{nonclean}} = 0.029$), while the less cleanest was TDC AqSolDB ($f_{\text{nonclean}} = 0.671$).

Candidate activity cliffs were taken from the precomputed audit outputs. These pairs were generated using a MoleculeACE-style[3] similarity screen based on molecular ECFP Tanimoto similarity, generic Murcko-scaffold ECFP Tanimoto similarity, and normalised SMILES Levenshtein similarity. Classification cliffs were defined by discordant class labels, whereas regression cliffs were defined by large absolute label differences. For the final SI figure, exact molecular matches were removed by excluding all pairs with molecular Tanimoto similarity $T = 1.0$. The remaining pairs were ranked by molecular Tanimoto similarity and then by absolute activity difference, and the top 10 non-exact cliffs were shown for each dataset.

After removing exact matches, the selected BBBP cliffs had $T = 0.79$–0.87 with a one-class label change in all cases. The selected AqSolDB cliffs had $T = 0.74$–0.94 and substantially larger absolute label differences ($\Delta y = 7.71$–10.33). This comparison illustrates (Fig. S5) that the less cleanest dataset contains high-similarity analogues with much larger apparent activity discrepancies, consistent with stronger activity-cliff and label-quality concerns.

Impact of synthetic dataset noise on regression performance

To assess how systematic dataset artefacts affect benchmark performance, we performed a controlled noise-injection study on seven Polaris regression datasets using a random forest regressor as a baseline (Fig. S6). Because the official Polaris test labels are private, we first re-split the available training data for each dataset into an internal training split and an internal held-out test split. Perturbations were then introduced at increasing fractions relative to the internal training-set size. For label conflicts and activity-cliff-like perturbations, only the internal training split was modified, while the held-out test split was kept fixed for evaluation. For the split-contamination scenario, controlled fractions of held-out test examples were leaked into the training data to mimic train–test overlap.

Across datasets, activity-cliff-like label flips and label conflicts led to a consistent degradation in predictive performance as the perturbation fraction increased. For example, in the cliffs scenario, the mean Pearson correlation decreased from 0.552 without added noise to 0.415 at a perturbation fraction of 0.1. In contrast, split contamination inflated performance rather than degrading it, consistent with the model rediscovering information that should have remained unseen during training. The waterfall summary further shows that the magnitude and direction of performance change depend strongly on the perturbation type. These results indicate that benchmark scores are sensitive to systematic data flaws, and that small differences between methods may reflect artefact sensitivity rather than genuine improvements in molecular property prediction.

Counterfactual benchmark-composition analysis

Purpose and scope

The main audit identifies contamination, label inconsistency, and chemical redundancy in benchmark splits, while the noise-injection experiment tests how models respond when such artefacts are deliberately inserted before retraining. The counterfactual

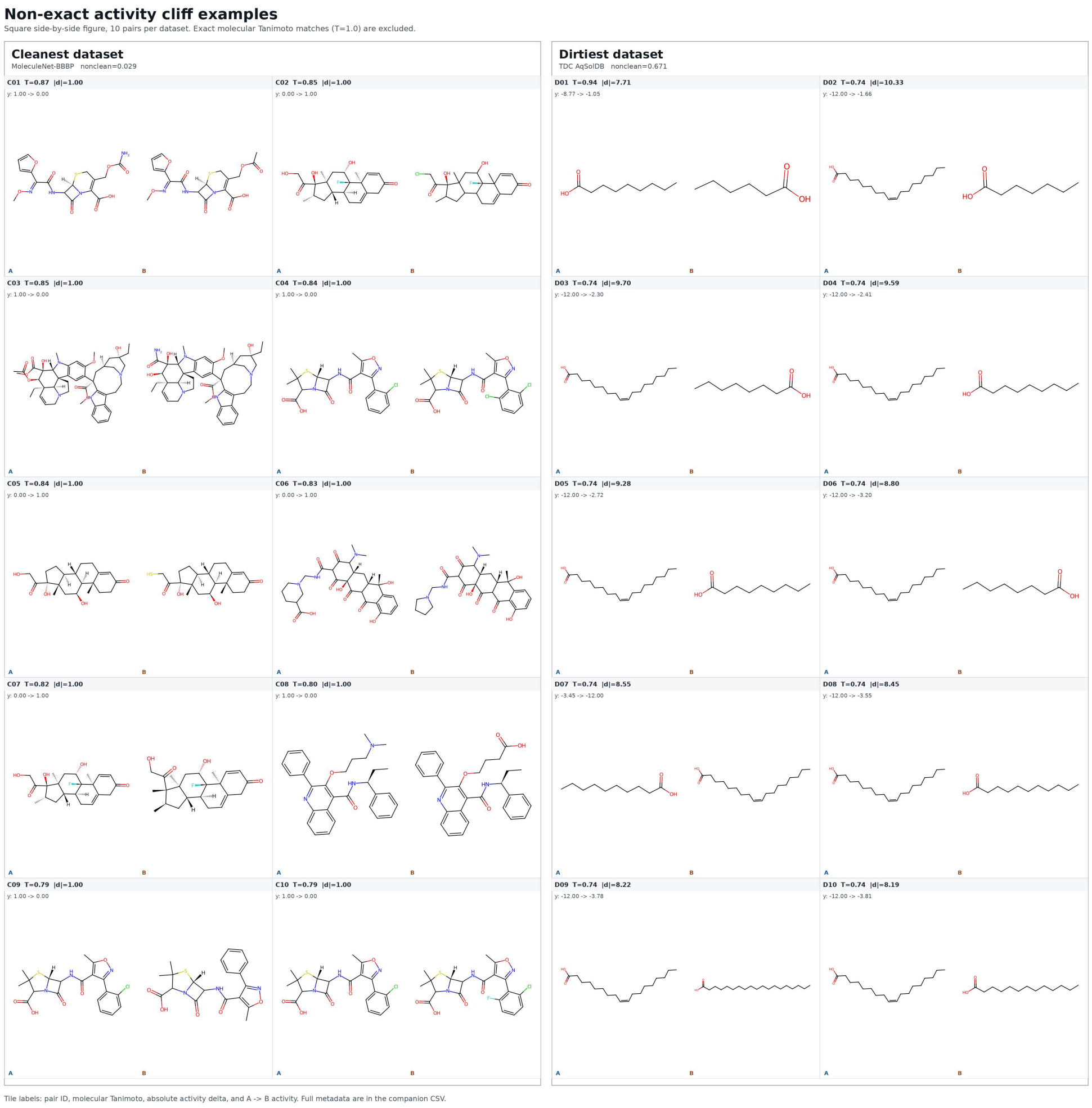

Fig. S5: **Side-by-side examples of non-exact activity cliffs from the cleanest and less cleanest audited datasets.** The cleanest dataset was MoleculeNet-BBBP ($f_{\mathrm{nonclean}} = 0.029$); the less cleanest was TDC AqSolDB ($f_{\mathrm{nonclean}} = 0.671$). Pairs with molecular Tanimoto similarity $T = 1.0$ were excluded, and remaining cliffs were ranked by molecular Tanimoto similarity and absolute activity difference. Each tile shows molecule A and molecule B, molecular Tanimoto similarity T, absolute activity difference $|\Delta|$, and the A-to-B activity change.

benchmark-composition analysis addresses a complementary post-hoc question: whether the leaderboard obtained from already trained models is stable when the composition of the evaluation set changes. The experiment therefore keeps trained-model predictions fixed and resamples only the held-out test molecules used for scoring. This isolates the effect of evaluation-panel composition on model metrics, ranks, and margins.

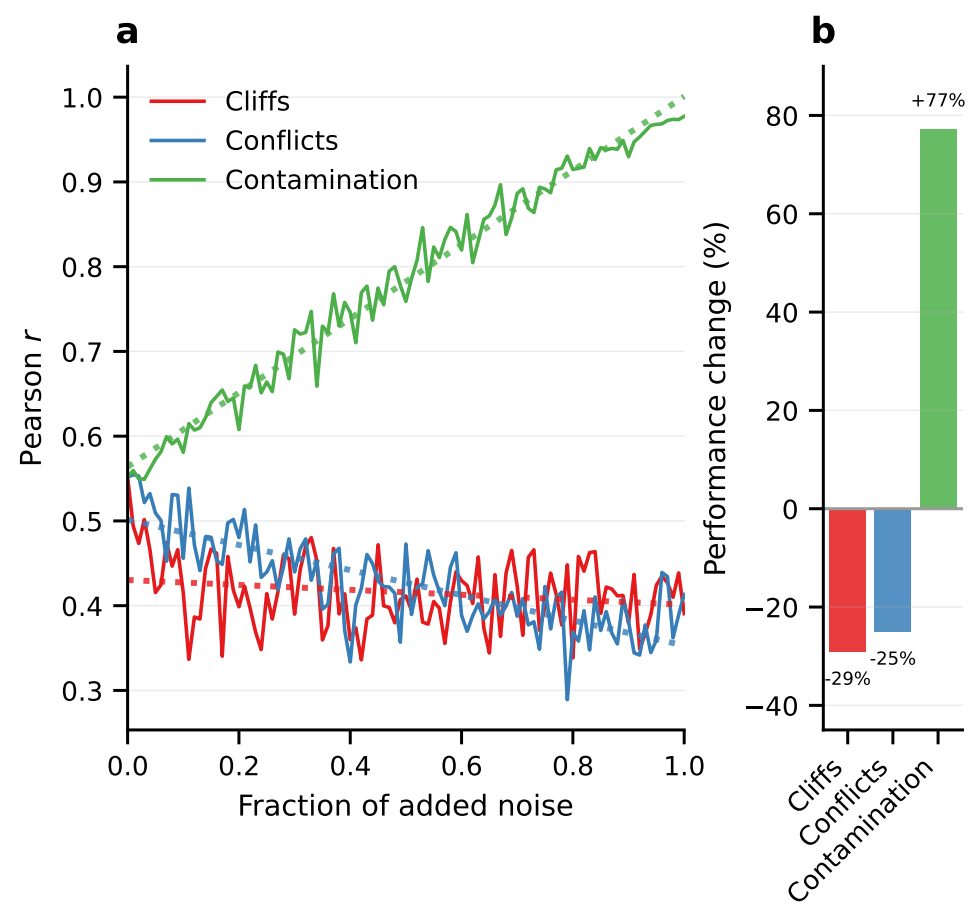

Fig. S6: **Noise-induced performance degradation across perturbation scenarios.** For each noise level and scenario, values are averaged across 7 Polaris benchmark datasets so each curve reflects the overall trend rather than a single dataset. A baseline random forest regressor was trained.[4] As the test set is private, the training data were re-split. **(a)** Pearson r is plotted as noise increases from 0 to 1 for cliffs, conflicts, and contamination (one fraction corresponds to the training set size). Solid lines show the averaged performance at each noise level, while dotted lines highlight the overall direction of change. **(b)** This is a waterfall-style summary, showing the total performance percentage change from no added noise to one fraction of added noise for each scenario.

The analysis was restricted to completed single-task molecular property datasets with finite held-out labels. The final set contained 28 datasets: 16 classification tasks evaluated with receiver operating characteristic area under curve (ROC AUC) and 12 regression tasks evaluated with root mean squared error (RMSE). Drug–target interaction (DTI) datasets were excluded because ligand–target pairs require a separate definition of benchmark composition, multitask datasets were skipped, and datasets without finite test labels could not be evaluated.

Audit labels used for panel construction

Each test molecule was assigned chemistry-audit annotations from the original train/test split. Exact train–test molecular identity leakage was defined as the same standardised molecule appearing in both train and test. Duplicate or conflicting labels were defined on repeated standardised molecules with inconsistent labels. For classification, any disagreement among labels for the same molecule was treated as a conflict; for regression, conflicts were defined by a prespecified label-difference threshold. Near-train analogue status was based on maximum extended-connectivity fingerprint (ECFP)/Tanimoto similarity to the training set, using a primary threshold of 0.85. Same-scaffold overlap was defined by Murcko-scaffold overlap with the training set.

For mutually exclusive audit groups, test rows were assigned in the following order: label conflict, exact train–test leak, near-train analogue, same scaffold, and audit-clean. This ordering keeps true contamination separate from analogue-rich evaluation cases. Exact leaks and label conflicts are treated as contamination. Near-train analogues and same-scaffold examples are not automatically erroneous; they represent local interpolation within familiar chemical neighbourhoods and can be relevant for lead-optimisation-style questions. Activity cliffs were intentionally not used as contamination flags.

Across all evaluated test rows, the mutually exclusive audit composition was 59.2 % audit-clean, 31.5 % same-scaffold overlap, 7.1 % near-train analogue, 1.3 % exact train–test leak, and 0.9 % label conflict. In the non-exclusive flag view, exact train–test molecular leakage affected 2.1 % of test rows, near-train analogue status affected 9.2 %, and same-scaffold overlap affected 37.3 %.

Counterfactual panel generation

Counterfactual panels were sampled from the original test set without duplicating a molecule within a panel. Across panels, molecules could be reused. For classification tasks, sampling preserved the label distribution where feasible. For regression tasks, labels were binned into five quantiles and panels were stratified by these bins where feasible. If a requested target composition could not be sampled from the available test rows, that composition was skipped.

Five panel types were generated. Clean-reference panels sampled only audit-clean test molecules. Observed-composition panels sampled according to the empirical audit-group proportions in the original test set. Exact/near-train-enriched panels controlled the fraction of examples that were either exact train–test leaks or near-train analogues, while excluding label conflicts. Conflict-enriched panels controlled the fraction of label-conflicting examples while excluding exact leaks where feasible. Matched random-control panels used the same panel sizes and target-rate schedule as the exact/near-train and conflict panels, but sampled randomly from eligible test rows.

The target rates were $0, 0.05, 0.10, 0.25$, the observed rate, 0.50, and 0.75, with 1000 panels generated for each feasible mode and target-rate combination. When panel size was set automatically, the largest feasible size for the requested compositions was used.

Metrics and rank stability

For every panel, model metrics and ranks were recomputed from the fixed held-out predictions. Rank 1 denotes the best model for the selected metric. The original full-test leader was defined as the best-ranked model on the complete original test set. Rank-1 probability is the fraction of counterfactual panels in which that original leader remained rank 1. SOTA-versus-baseline margins were computed against the random forest (RF) baseline, with signs normalised so that positive values always indicate an advantage for the original leading model, including for lower-is-better regression metrics.

Kendall's τ was used to compare each counterfactual-panel leaderboard with the original full-test leaderboard. This statistic is appropriate because the relevant scientific claim is ordinal: which model ranks above which. It is also comparable across

classification and regression tasks because it does not depend on the scale of the underlying metric. $\tau = 1$ indicates identical model ordering, $\tau = 0$ indicates no ordinal association, and lower values indicate increasing numbers of pairwise rank reversals. Empirical 2.5 %–97.5 % intervals were computed over panels.

Cross-dataset summary

The original full-test leader was advanced LightGBM in 15 of 28 datasets, RF in 7, basic LightGBM in 3, the basic multilayer perceptron (MLP) in 2, and linear ECFP in 1. On audit-clean panels, the original leader remained rank 1 in 23 of 28 datasets, with a mean rank-1 probability of 0.82. This indicates that the original leader was usually not explained solely by exact train–test identity leakage or obvious label conflicts.

At the observed exact/near-train composition, the original leader's rank-1 probability fell below 0.5 in 15 of 28 datasets, and the mean Kendall τ to the original leaderboard was 0.54. When exact/near-train examples were enriched to 75 %, the original leader's rank-1 probability fell below 0.5 in 20 of 28 datasets, and mean Kendall τ was 0.47. Matched random-control panels were also unstable, indicating that finite evaluation-panel size and ordinary resampling contribute to rank fragility.

The clearest audit-specific signal came from label conflicts. Conflict-enriched panels were feasible in 14 datasets. At the observed conflict composition, the original leader's rank-1 probability fell below 0.5 in 12 of these datasets, and mean Kendall τ dropped to 0.33. At 75 % conflict composition, the rank-1 probability fell below 0.5 in 13 of 14 datasets. This supports the conclusion that leaderboard claims should be reported together with audit-clean, conflict-controlled, and analogue-controlled stability checks.

References

[1] Mendez, D. *et al.* ChEMBL: Towards direct deposition of bioassay data. *Nucleic acids research* **47**, D930–D940 (2019).

[2] Li, J. *et al.* Leak Proof PDBBind: A Reorganized Data Set of Protein–Ligand Complexes for More Generalizable Binding Affinity Prediction **130**, 730–740. URL https://doi.org/10.1021/acs.jpcb.5c08598.

[3] van Tilborg, D., Alenicheva, A. & Grisoni, F. Exposing the Limitations of Molecular Machine Learning with Activity Cliffs. *Journal of Chemical Information and Modeling* **62**, 5938–5951 (2022).

[4] Pedregosa, F. *et al.* Scikit-learn: Machine Learning in Python. *Journal of Machine Learning Research* **12**, 2825–2830 (2011).
